# eIF6 coordinates insulin sensitivity and lipid metabolism by coupling translation to transcription

**DOI:** 10.1038/ncomms9261

**Published:** 2015-09-18

**Authors:** Daniela Brina, Annarita Miluzio, Sara Ricciardi, Kim Clarke, Peter K. Davidsen, Gabriella Viero, Toma Tebaldi, Nina Offenhäuser, Jan Rozman, Birgit Rathkolb, Susanne Neschen, Martin Klingenspor, Eckhard Wolf, Valerie Gailus-Durner, Helmut Fuchs, Martin Hrabe de Angelis, Alessandro Quattrone, Francesco Falciani, Stefano Biffo

**Affiliations:** 1 INGM, ‘Romeo ed Enrica Invernizzi', 20122 Milano, Italy; 2 Centre for Computational Biology and Modeling, Institute of Integrative Biology, University of Liverpool, Liverpool L69 7ZB, UK; 3 Institute of Biophysics, 38123 Trento, Italy; 4 Centre for Integrative Biology, University of Trento, 38123 Trento, Italy; 5 IFOM Foundation, 20139 Milano, Italy; 6 German Mouse Clinic, Institute of Experimental Genetics, Helmholtz Center Munich, 85764 Neuherberg, Germany; 7 Institute of Molecular Animal Breeding and Biotechnology, Gene Center, Ludwig-Maximilian-University, 81377 Munich, Germany; 8 Else Kröner-Fresenius Center, Technische Universität München, 85354 Freising, Germany; 9 Center of Life and Food Sciences Weihenstephan, Technische Universität München, 85354 Freising, Germany; 10 German Center for Diabetes Research, 85764 Neuherberg, Germany; 11 Department of Biosciences, University of Milan, 20133 Milan, Italy

## Abstract

Insulin regulates glycaemia, lipogenesis and increases mRNA translation. Cells with reduced eukaryotic initiation factor 6 (eIF6) do not increase translation in response to insulin. The role of insulin-regulated translation is unknown. Here we show that reduction of insulin-regulated translation in mice heterozygous for eIF6 results in normal glycaemia, but less blood cholesterol and triglycerides. eIF6 controls fatty acid synthesis and glycolysis in a cell autonomous fashion. eIF6 acts by exerting translational control of adipogenic transcription factors like C/EBPβ, C/EBPδ and ATF4 that have G/C rich or uORF sequences in their 5′ UTR. The outcome of the translational activation by eIF6 is a reshaping of gene expression with increased levels of lipogenic and glycolytic enzymes. Finally, eIF6 levels modulate histone acetylation and amounts of rate-limiting fatty acid synthase (Fasn) mRNA. Since obesity, type 2 diabetes, and cancer require a Fasn-driven lipogenic state, we propose that eIF6 could be a therapeutic target for these diseases.

## Abstract (web summary)

Insulin enhances mRNA translation via the translation initiation factor eIF6. Here, Brina *et al*. show that insulin-mediated activation of eIF6 is associated with the selective translation of genes involved in glycolysis and lipid synthesis with characteristic G/C-rich and uORF sequences in their mRNA.

## Graphical abstract

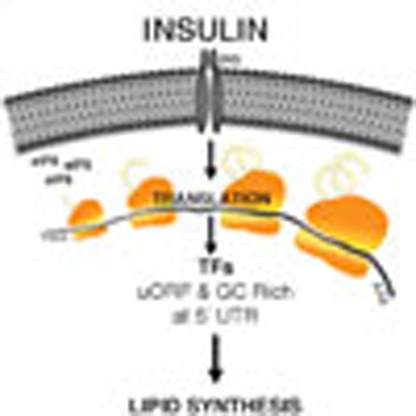

Insulin controls glycaemia. Insulin resistance, the lack of responsiveness to insulin, is at the basis of the pathogenesis of many metabolic disorders, widespread in the world population and constantly growing^1^. Nonalcoholic fatty liver disease is the most common liver disorder in the Western world^2^ and is universally associated with hepatic insulin resistance, which increases the risk of impaired control of fasting glycaemia and of type 2 diabetes^3^. The understanding of the molecular mechanism of insulin action provides a tool to optimize strategies to overcome insulin resistance.

Insulin induces a multifocal response that includes glucose uptake, signalling to the transcriptional machinery and an increase in translation^4^. It is well know how insulin transmit signals to the translational machinery^5^. Briefly, mTOR kinase is a central player in translation^6^. Insulin stimulates mTORc1 (complex I) and mTORc2 (complex 2) activity. mTORc1 regulates the formation of the cap complex eIF4F and it phosphorylates 4E-BPs, releasing the cap complex binding protein eIF4E (ref. 7). Free eIF4E assembles in the eIF4F complex, which contains mRNA, eIF4A helicase and eIF4G. The eIF4F complex binds 43S ribosomal subunits leading to the formation of 48S preinitiation complexes and subsequent activation of cap-dependent translation. Therefore, the insulin-mTORc1-eIF4F axis controls the translational efficiency of specific mRNAs downstream of mTORc1 activity. In the context of insulin, inactivation of regulators 4E-BP1 and 4E-BP2 causes insulin resistance^8^, and treatment with rapamycin and its analogues (rapalogues) generates glucose intolerance^9^ or even the onset of insulin resistance^10^. These data leave unresolved the issue on the druggability of the translational apparatus in metabolic syndromes. In cycling cells, mTOR activation results in the induction of cell growth and cell cycle progression^7^. Consistently, inhibition of mTORc1 dependent translation by rapalogs has an anti-neoplastic effect in selective cancers with PI3K-mTOR activation^11^,^12^.

The RAS/MAPK pathway converges also on translation^7^. Cancer patients with RAS mutations are insensitive to mTORc1 inhibition^13^. The insensitivity to rapamycin inhibition of cells with mutated RAS suggests that other initiation factors act independently from mTOR among them, eIF4E and eIF6. eIF4E phosphorylation is regulated by the RAS-Mnk1/2 cascade and, in the context of cancer, constitutive eIF4E activation is associated with tumour progression^14^,^15^. eIF6 is an initiation factor acting independently from mTOR^16^. eIF6 heterozygosity restrains tumourigenesis and leads to a striking increased survival in a mouse model of Eμ-Myc lymphoma^17^. eIF6 amplification occurs in luminal breast cancer^18^. eIF6 is an anti-association factor that prevent formation of inactive 80S complex, by binding to 60S (ref. 19). eIF6 phosphorylation is stimulated by phorbol esters inducing protein kinase C (PKC) activation and in response to insulin^20^. The C terminus of eIF6 has multiple phosphorylation sites including Ser235 (ref. 21). Normal eIF6 sustains translation, whereas a Ser235Ala mutant does not^22^. The protective effects of eIF6 depletion on tumour growth and the specific action of eIF6 on insulin-stimulated translation suggest that eIF6 and 60S availability control the translation of specific mRNAs. eIF6 has been reported to regulate miRNA-based repression, but this role has not been confirmed by further studies^23^. Thus eIF6 targets remain unknown.

Driver regulatory *cis*-sequences in the 5′ untranslated region (UTR) or 3′ UTR of mRNAs may account for differential translation. Growth factors stimulate the translation of 5′structured or G/C containing mRNAs that encode for proteins necessary for cell cycle progression^24^ and/or of terminal oligopyrimidine tract (TOP) mRNAs encoding for ribosomal proteins^25^,^26^ or pyrimidine-rich translational elements^25^. Cellular Internal Ribosome Entry Site may sustain translation in the absence of nutrients^27^. Upstream open reading frame (uORF) sequences regulate translation in conditions of amino-acid deprivation, dsRNA infection, unfolded protein response (UPR)^28^. Loss of eIF4F integrity by eIF4G depletion has been shown to affect polysomal association of uORF-containing mRNAs, suggesting that uORFs might have other functions, independent from amino-acid deprivation or UPR^29^.

In conclusion several questions arise: (i) what is the physiological action of the insulin-eIF6 axis? (ii) are specific mRNAs preferentially translated upon activation of eIF6? (iii) is eIF6 targetable? To address these questions we combined analysis on mouse, human and cellular models. We found that eIF6 translational activity is necessary to co-ordinate an integrated translational response that shapes the metabolism of cells. We show that eIF6 is an initiation factor that activates *de novo* lipogenesis and affects insulin sensitivity. Our data support a model in which eIF6-driven translation reinforces insulin-stimulated lipid synthesis and may become a therapeutic target in metabolic syndromes.

## Results

### eIF6 is required for efficient lipid synthesis *in vivo*

**Figure 1:**
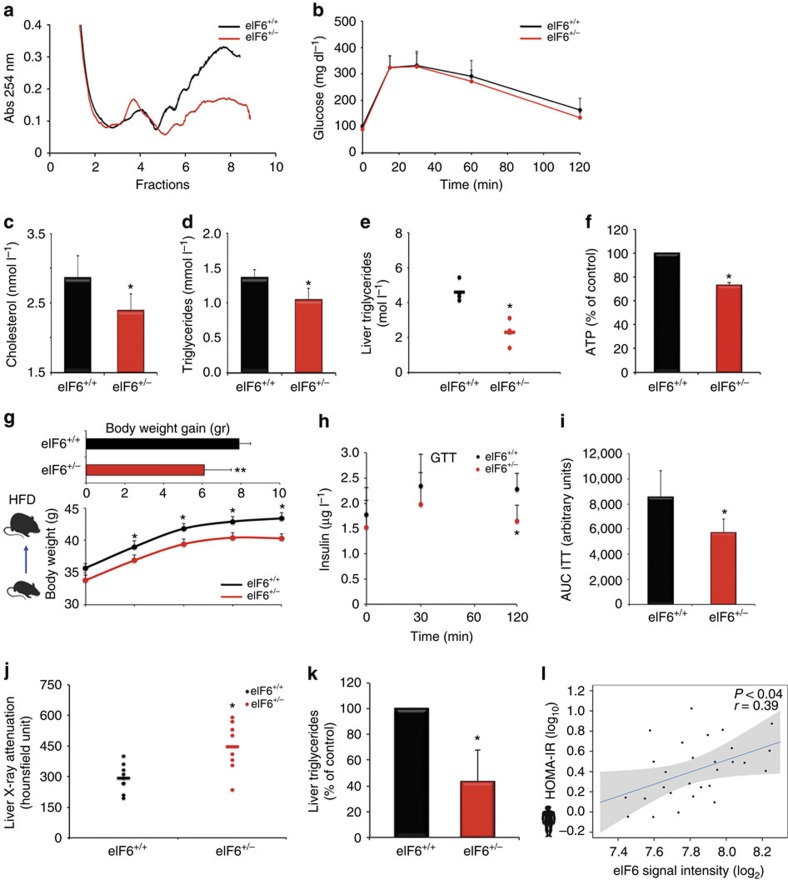
eIF6 depletion impairs polysome formation in the liver after feeding, reduces lipidemia, protects from HFD and liver steatosis. All graphs, black, wt; red, eIF6 het mice (**a**) Representative liver polysomal profiles of wt (black) and eIF6 het (red, +/−) show reduced polysomes in het after overnight fasting and 4 h refeeding. Experiment repeated at least five times. (**b**) GTT is normal in eIF6 het (red) compared with wt. *N*=7. (**c**,**d**) Cholesterol and triglycerides levels are reduced in blood of eIF6 het compared with wt ones (*N*=10). (**e**) Liver triglyceride content is reduced in eIF6 het mice (*N*=4). (**f**) ATP liver content is reduced in eIF6 het. *N*=3. (**g**) eIF6 het mice are protected from HFD: body weight gain is reduced in eIF6 het mice compared with wt. *N*=10. Top bars show absolute body weight gain. Lower graph, body weight. (**h**) GTT eIF6 het mice have lower insulinaemia at 2 h after glucose injection. *N*=10. (**i**) ITT (AUC ITT, area under curve) shows partial amelioration of diet-induced insulin resistance. *N*=5 (**j**,**k**). Increased liver-X-ray attenuation (*N*=8) indicating reduced fat deposition in the liver (**j**) confirmed by autoptic liver triglyceride content on separate animals (*N*=5) (**k**). (**i**) In humans, eIF6 levels inversely correlate with insulin sensitivity, as measured by HOMA-IR. In all panels, data are represented as mean±s.d. Statistical *P* values were calculated by two-tailed *t*-test (*P value ≤0.05).

eIF6 heterozygous (het) cells express 50% eIF6 compared with wild type (wt), have normal basal translation, but impaired insulin-stimulated translation^22^. eIF6 het mice are healthy, leaner^22^ and resistant to Myc-induced lymphomagenesis compared with wt ones^17^. We employed a cohort of wt and het mice, *n*=40, in a pure C57BL/6 genetic background (Supplementary Fig. 1a,b). We analysed liver polysome peaks as an indicator of initiation of translation, *in vivo*, after feeding. Polysomes from eIF6 het mice were lower than in wt, consistent with reduced initiation of translation, *in vivo* (Fig. 1a) after feeding. During fasting, eIF6 het mice displayed a polysome profile similar to wt ones (Supplementary Fig. 1c). This model therefore allows the analysis of the physiological role of translation *in vivo*, after feeding. We first asked whether glycemic control is maintained in conditions of eIF6 deficiency. To evaluate glucose tolerance, we performed a glucose tolerance test (GTT). We found that eIF6 het mice had normal response to glucose load (Fig. 1b). A broad clinical chemistry analysis revealed that in refed conditions eIF6 het mice had reduced blood cholesterol levels (Fig. 1c), triglycerides (Fig. 1d), liver triglycerydes (Fig. 1e), non-essential fatty acids (Supplementary Fig. 1d). Glycaemia was slightly lower in both fasted and refed conditions (Supplementary Fig. 1d). Insulin levels were lower in fasting conditions (Supplementary Fig. 1d). All other parameters in fasting were identical between wt and het mice (Supplementary Fig. 1d). Markers for hepatic function were identical between wt and het mice, suggesting overall normal hepatic function (Supplementary Fig. 1e). In liver, insulin stimulates glucose storage through stimulation of glycogen synthesis. We found that eIF6 het had glycogen levels identical to wt mice (Supplementary Fig. 1f,g). Liver ATP levels were lower in eIF6 het mice compared to wt (Fig. 1f). In conclusion, eIF6 mice have impaired insulin-stimulated translation^22^ accompanied by reduction of blood and liver lipid levels without impairment of glucose blood control and glycogen synthesis.

To define whether the translational deficit on insulin stimulation has an impact in pathological conditions, we tested the response of eIF6 het mice to a high-fat diet (HFD) regimen: we found that eIF6 het mice had a reduced weight gain during HFD compared with wt controls (Fig. 1g) but no substantial difference in glucose tolerance (Supplementary Fig. 1h) and insulin levels at time 0 (Supplementary Fig. 1i). Nonetheless, insulin levels were significantly lower in het mice compared with wt mice after 120 min from glucose injection (Fig. 1h). However, an insulin tolerance test (ITT) showed that eIF6 het mice responded better to insulin (Fig. 1i; Supplementary Fig. 1j). Following a HFD, eIF6 het mice showed increased liver-X-ray attenuation and decreased triglycerides (TG) levels compared with wt mice (Fig. 1j,k) demonstrating reduced hepatic lipid content. At autopsy, eIF6 het livers were less enlarged (Supplementary Fig. 1k). We analysed whether a relationship between eIF6 levels and insulin resistance, could be found in humans. The homoeostasis model assessment of insulin resistance (HOMA-IR) is an established measure of whole-body insulin sensitivity, the higher the value the more insulin resistant. We data-mined the relationship between subcutaneous fat expression levels of eIF6 and HOMA-IR. We found (Fig. 1l) that eIF6 expression negatively correlates with insulin sensitivity, as an independent factor (Fig. 1l).

In conclusion, our data demonstrate that the impairment of translation due to eIF6 depletion affects liver lipid metabolism leading to reduced susceptibility to hepatic lipid accumulation.

### eIF6 stimulates fatty acid synthesis cell autonomously

**Figure 2:**
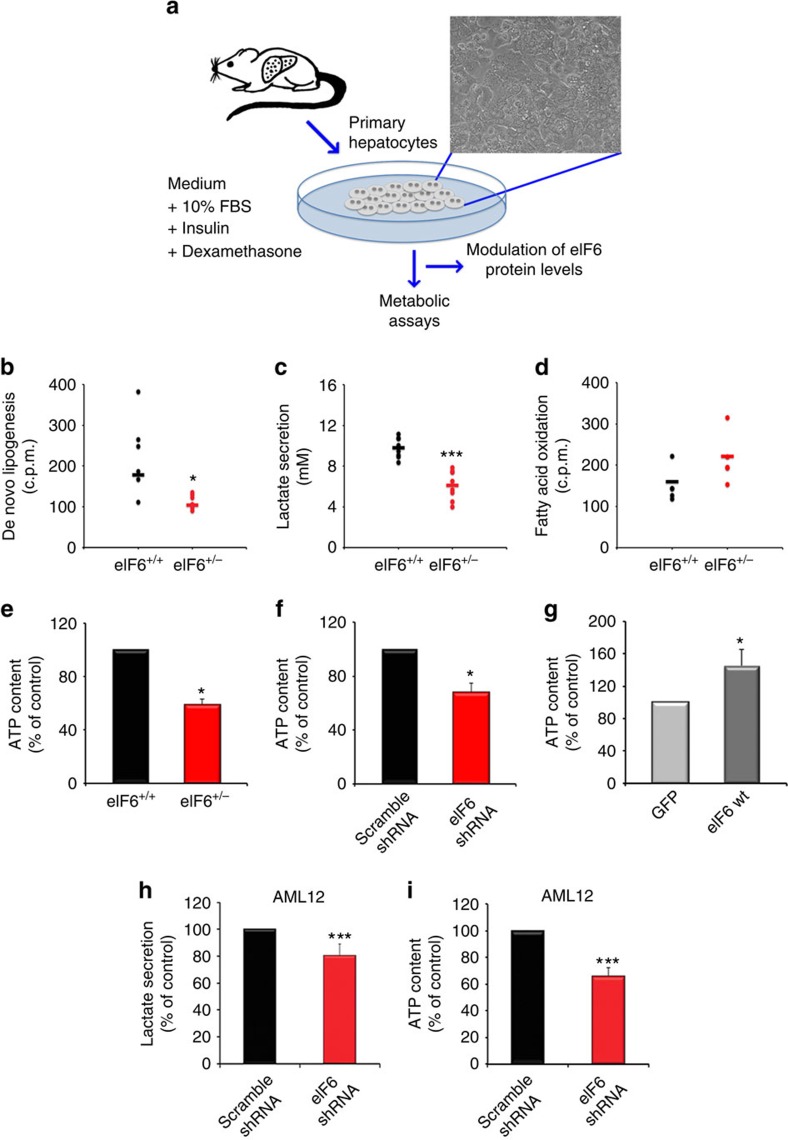
eIF6 activity cell autonomously controls fatty acid synthesis, glycolysis and ATP levels. (**a**) Outline of the analysis. Primary hepatocytes from mice were isolated and assayed as specified. Experiments performed on biological replicates. All data are expressed as percentage of controls (wt), analysing primary cells from littermate couples of mice. (**b**) *De novo* lipogenesis, measured by labelling with D-[6-^14^C]-glucose and subsequent fatty acid analysis, is reduced in cells from eIF6 het mice. Cells were kept in 100 nM insulin and 20 mM glucose. *N*=6 (**c**) Lactate secretion, an index of glycolysis flux, is reduced in hepatocytes from eIF6 het mice. Basal values are around 30 pm μg^−1^ proteins per hour. *N*=6. (**d**) Fatty acid oxidation is not significantly affected (basal values of control around 1,000 c.p.m. μg^−1^ proteins). *N*=4 (**e**–**g**) ATP content depends from eIF6 levels: (**e**) ATP decreased in eIF6 het mice compared with wt ones. (**f**) Acute depletion of eIF6 leads to a reduction of ATP levels in eIF6^+/+^ cells, whereas (**g**) restoration of eIF6 levels leads to an increase in ATP in eIF6^+/−^ cells. (**h**) Lactate secretion as in (**f**) after eIF6 shRNA in AML12 cells. (**i**) ATP as in (**d**), after eIF6 shRNA in AML12 cells. In all panels, data are represented as mean±s.d. Statistical *P* values were calculated by two-tailed *t*-test (*P value ≤0.05, ***P≤0.001). (**e**–**i**), *N*=3.

Changes in blood cholesterol and in ATP liver levels raise the question whether the metabolic changes driven by eIF6 are cell autonomous or due to indirect systemic events such as, for instance, reduced food intake/absorption or increased basal metabolic rate. We analysed metabolic parameters from primary hepatocytes isolated from mice (Fig. 2a). eIF6 het hepatocytes have 50% eIF6 protein compared with wt (Supplementary Fig. 2a), and, consistent with the *in vivo* phenotype, they fail to increase protein synthesis after insulin stimulation (Supplementary Fig. 2b). Glucose uptake of eIF6 het cells was identical to wt, consistent with the normal GTT observed *in vivo* (Supplementary Fig. 2c). Glucose uptake was also normal in eIF6-depleted 3T3-L1 cells (Supplementary Fig. 2d). Notably, primary eIF6 het hepatocytes, compared with wt ones, showed a significant reduction of *de novo* lipogenesis (Fig. 2b). Insulin stimulates glycolysis. We found that primary het hepatocytes had a significant reduction of glycolysis, as measured by lactate secretion (Fig. 2c). Fatty acid oxidation under feeding (Fig. 2d) was not significantly changed between wt and het. Hepatocytes from eIF6 het mice also had a reduction in ATP concentration (Fig. 2e). These results mirror the liver condition, *in vivo*, as described in Fig. 1. To investigate if eIF6 acutely controlled ATP levels, we either silenced eIF6 by lentiviral-mediated shRNA in wt hepatocytes or re-expressed eIF6 by lentiviral-mediated infection in het hepatocytes. Briefly, we found that, in wt hepatocytes, eIF6 acute depletion reduced intracellular ATP (Fig. 2f), whereas eIF6 overexpression, in het ones, increased it (Fig. 2g). We asked whether eIF6 might steer the metabolic activity of established cell lines. AML12 cells are derived from non-tumourigenic murine liver hepatocytes. In AML12 cells, we found that depletion of eIF6 by shRNA reduced glycolysis, measured as lactate secretion (Fig. 2h), and ATP content (Fig. 2i).

The mTORc1 pathway is involved in the response to nutrients and growth factors. We tested whether eIF6 acts upstream of mTORc1 activation. Modulation of eIF6 levels did not affect phosphorylation of rpS6 at Ser235, a substrate of mTORc1-S6K cascade, and 4E-BP1, a substrate of mTORc1 (Supplementary Fig. 2e). Taken together data demonstrate that eIF6 translational activity steers the metabolism of cells in a cell autonomous fashion. Specifically, eIF6 induces glycolysis and fatty acid synthesis.

### Insulin-eIF6 modulate translation of lipogenic factors

**Figure 3:**
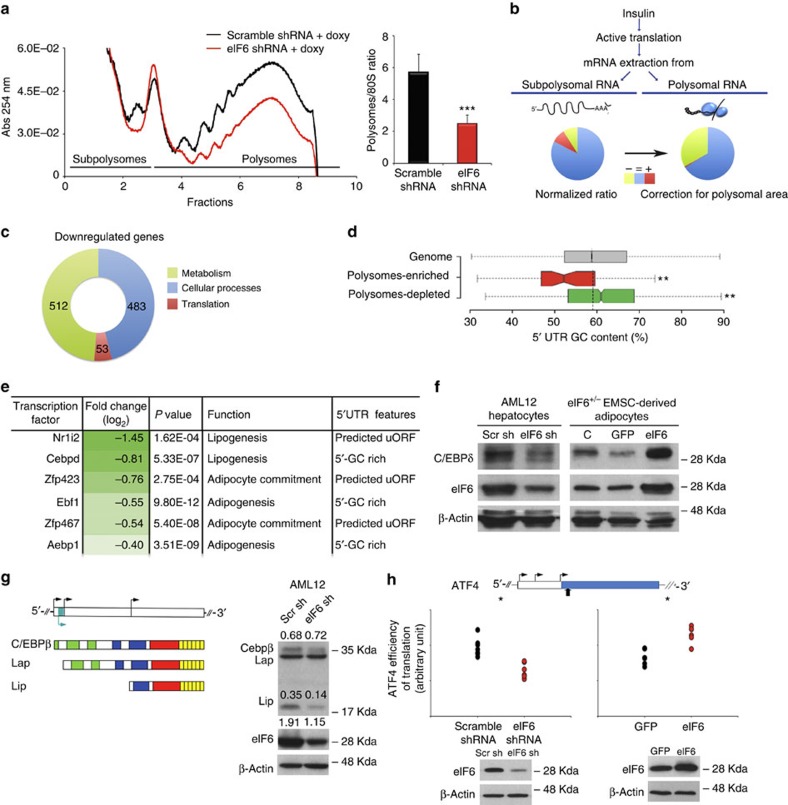
eIF6 translational activity controls the expression of lipogenic transcription factors. (**a**) Global initiation is reduced on eIF6 inhibition and insulin administration, as shown by polysome/80S ratio. Data are represented as mean±s.d. Statistical *P* values were calculated by two-tailed *t*-test (*n*=4. ***P value ≤0.001). (**b**) Outline of the analysis and quantitative results: a subset of mRNA is differentially depleted (green) from eIF6 shRNA polysomes. The normalized ratio was obtained by the quantile method. Correction taking in account the reduction of polysomal area shows that more genes are affected by eIF6 deficiency. (**c**) Gene Ontology analysis shows that metabolism predominates in eIF6-affected genes, from the normalized ratio pool (*P* value ≤10^−12^). (**d**) Structural 5′-UTR analysis of genes affected by eIF6 deficiency. Polysomes from eIF6-depleted cells show reduction of G/C rich 5′ mRNAs (green box) (**e**) Transcription factors involved in lipogenesis are depleted from polysomes upon eIF6 shRNA. Structural features of their 5′ UTR are included. (**f**) C/EBPδ protein expression is modulated by eIF6. eIF6 increase, by lentiviral administration, induces C/EBPδ. eIF6 depletion, by lentiviral-mediated shRNA, reduces C/EBPδ. Representative experiments done in triplicates on AML12 hepatocytes and reconstituted mesenchymal stem cells (**g**) Lipogenic transcription factor C/EBPβ (LIP isoform) is reduced in eIF6-depleted cells. Left, mRNA structure of C/EBPβ generating three isoforms by differential translation (arrows: translation start sites). Blue small box with blue arrows is the C/EBP uORF. Right panel show a blot with LAP, LIP and C/EBPβ isoforms: LIP isoform is specifically downregulated. (Representative experiment done in triplicates). (**h**) ATF4 reporter translation depends from eIF6. Reporter assay of reinitiation with luciferase (blue box) cloned downstream of natural ATF4 uORF. eIF6 upregulation increases ATF4 translational reporter activity, eIF6 downregulation decreases it. Data are normalized on translation of a cap-dependent firefly control. The large black arrow represents the stop codon of the second uORF. Small arrows are start codons. Blue box is the reporter. Statistical *P* values were calculated by two-tailed *t*-test, as above (*P value ≤0.05; **P≤0.01; ***P≤0.001).

Data from Figs 1 and 2 suggest that eIF6 drives the translation of specific factors that regulate a lipogenic switch. We analysed how eIF6 modulates insulin-stimulated translation by using a fast doxycycline-inducible system, combined with polysomal microarray. Forty-eight hours after doxycycline treatment, AML12 cells with inducible eIF6 shRNA presented a reduction of eIF6 (Supplementary Fig. 3a) and resistance to insulin-stimulated methionine incorporation (Supplementary Fig. 3b). Polysomal analysis of insulin-stimulated cells showed that eIF6 downregulation reduced the polysome/80S ratio as expected by the loss of eIF6 anti-association activity (Fig. 3a). In unstimulated conditions eIF6 deficiency did not affect polysome peaks (Supplementary Fig. 3c).

Next, we analysed mRNA from either polysomes or subpolysomes of insulin-stimulated cells by microarray analysis, (*n*=4; Fig. 3b; Supplementary Data 1). The analysis showed that eIF6 depletion induced a decrease in the polysomal occupancy of more than 1,000 mRNAs (Fig. 3b). Moreover, after correcting for polysome occupation, that is, absolute number of polysome associated mRNAs, we found that up to 1/3 of genes expressed in AML12 cells were inhibited by eIF6 depletion (5312/15405; Fig. 3b). We conclude that a large subset of mRNAs, ≈1,000, is strongly inhibited, under conditions of insulin stimulation and depletion of eIF6. These mRNAs were further analysed.

Gene ontology analysis of strongly inhibited mRNAs showed that they participated to metabolic processes and translation (Fig. 3c). Notably, sterol regulatory element binding protein 1 (SREBP1), a transcription factor controlling glucose metabolism and fatty acid production, modulated by mTOR (ref. 30), was not found regulated at the polysomal level by eIF6 (Supplementary Data 1). Sequence analysis demonstrated that the most affected mRNAs inhibited by eIF6 depletion contained G/C rich regions in their 5′ UTR (Fig. 3d). Several transcription factors involved in lipid synthesis were in the group of G/C rich mRNAs downregulated at the polysomal level by eIF6 depletion (Fig. 3e). By contrast, mRNAs with less G/C content competed more favourably onto polysomes (Fig. 3d), in the absence of eIF6. Next, we tested the relevance of polysomal predictions by quantifying, for some adipogenic transcription factors, the eIF6-based regulation at the protein level. C/EBPδ (ref. 31) is an adipogenic transcription factor^32^. We found that eIF6 positively regulates C/EBPδ protein, as predicted by polysomal abundance, that is, eIF6 depletion reduces C/EBPδ protein and eIF6 upregulation increases it (Fig. 3f). In the same conditions, C/EBPδ mRNA was downregulated on polysomes by eIF6 depletion (Supplementary Fig. 3d). Sequence analysis of lipogenic transcription factors downregulated on polysomes by eIF6 depletion showed also the presence of uORF-containing mRNAs. uORF repress translation, acting as insulators of the downstream ORF, unless released by either reinitiation or leaky scanning. Classically, uORF have been associated to sensing stress, including amino-acid deficiency^33^,^34^. Our experiments suggest that transcription factors containing uORF may be (also) involved in lipid biosynthesis. We tested two transcription factors containing established uORF sequences, C/EBPβ and ATF4. C/EBPβ regulates adipogenesis^35^. C/EBPβ mRNA generates, through alternative translation three isoforms, LAP* (C/EBPβ), LAP and LIP (ref. 36). eIF6 shRNA downregulated C/EBPβ mRNA polysomal occupancy (Supplementary Fig. 3e). In the same condition, we observed that LIP translation product of C/EBPβ was consistently downregulated, whereas LAP was not (Figs 3g and 5e). ATF4 is a classical transcription factor with uORFs regulated at the translation level by reinitiation. ATF4 is robustly linked to the UPR^37^. However, the *in vivo* phenotype of ATF4 depletion resembles eIF6 knockout phenotype with lower cholesterolemia and resistance to fat accumulation^38^. Then, we used an ATF4 uORFs-containing mRNA upstream of a luciferase reporter, to specifically measure its translational efficiency in conditions of eIF6 modulation. We found that eIF6 depletion reduces ATF4 reporter activity and eIF6 overexpression increases it (Fig. 3h).

In conclusion, we demonstrate that reduced eIF6 blunts insulin-regulated translation of specific mRNAs regulating metabolism. Specifically, we find a robust change in the translational activation of multiple transcription factors involved in lipogenesis, which fits the data presented in Figs 1 and 2, and predicts further gene-expression modulation.

### eIF6 axis elicits a translation to transcription programme

**Figure 4:**
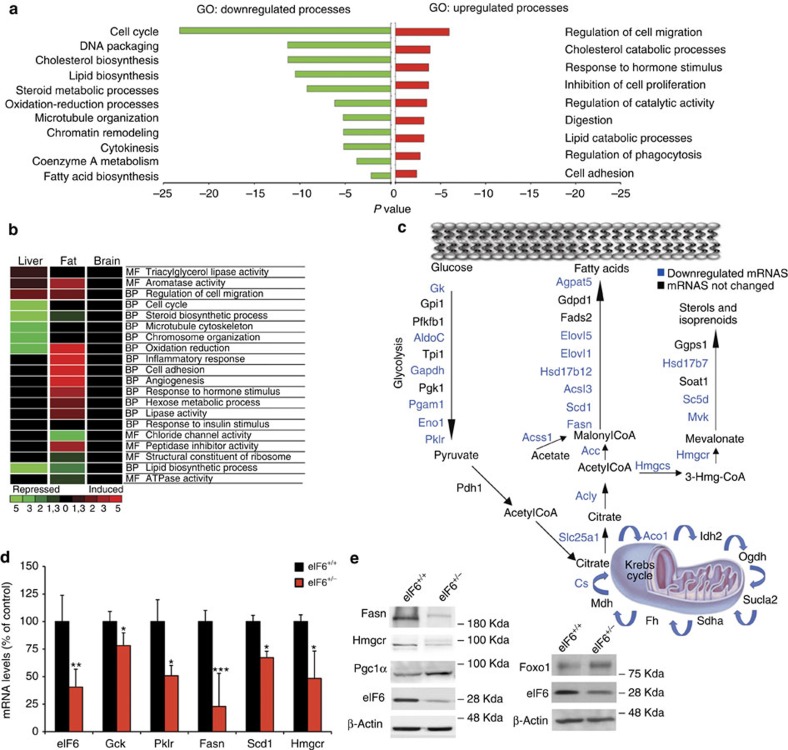
eIF6 depletion induces a specific transcriptional signature in insulin-responsive tissues. (**a**) Microarray analysis on adult livers show extensive remodelling of gene expression in eIF6 het mice. Co-ordinated transcriptional changes in eIF6 het livers identify a profound metabolic reprogramming, at the steady-state level, consistent with reduction of lipid biosynthesis, and additional effects on chromatin remodelling and cell cycle. (**b**) Microarray analysis on multiple organs. Heat map analysis of liver, fat and brain shows a common signature of inhibition of lipid biosynthetic processes in fat and liver but not in the brain. The scale represents statistical significance of the enrichment of Gene Ontology terms obtained by using a modified Fisher's exact test (EASE score). This produces a *P* value, which is then corrected for multiple testing using the FDR (Benjamini). 1.3=*P* value ≤0.05, 2=*P* value ≤0.01, 3=*P* value ≤0.001, 5=*P* value ≤0.00001 (**c**) Outlines of genes affected by eIF6 levels in the fatty acid synthesis, glycolysis and cholesterol synthesis pathway. All blue-labelled genes are downregulated at the transcriptional level. (**d**) Real-time analysis of selected targets validates all target genes (liver tissues). Here we show representative biological triplicates of experiments performed on separate cohorts of animals, (five for eIF6 and Fasn and two for the others) (data shown for *n*=3; mean±s.d.). (**e**) Consistent changes are also found at the protein level. Representative blots of Hmgcr, Fasn (downregulated at the mRNA level) and of Pgc-1 α (upregulated at the mRNA level), done in triplicates. See also Supplementary Fig. 4. (*n*=3).

The liver is a major insulin-responsive organ that responds to high nutrients and high insulin by the induction of protein synthesis. Driven by the observations that eIF6 regulates *de novo* lipogenesis and the translation of (some) lipogenic transcription factors, we aimed at defining the genes affected downstream. We simultaneously assessed steady-state mRNA levels and polysome associated mRNAs in liver of wt and het mice under feeding conditions (Supplementary Data 2). We describe for clarity only mRNAs whose levels change at the steady state (total).The gene-expression signature of eIF6 depletion, at the steady-state mRNA level, was consistent with the metabolic and rapid translational changes mastered by eIF6 activity, and extended them. Briefly, eIF6 depletion induced a peculiar gene-expression signature marked by the downregulation of mRNAs encoding for key proteins regulating cell cycle, DNA packaging, cholesterol and lipid biosynthesis and glycolysis (Fig. 4a,b; Supplementary Fig. 4a). The lipid biosynthetic programme was affected in liver and fat, but not in brain (Fig. 4b).

Gene ontology analysis demonstrated that all enzymes involved in fatty acid, sterol synthesis and glycolysis were significantly decreased more than twofold by eIF6 depletion (Fig. 4c). Among them, we found ATP cytrate lyase, acetyl-CoA carboxylase 1 and the rate-limiting enzymes fatty acid synthase (Fasn) involved in lipogenesis, elongation of long-chain fatty acids family member 6 and stearoyl-CoA desaturase 1 catalysing fatty acids elongation and desaturation step; Hmg-Coenzyme A synthase and Hmg-Coenzyme A reductase (Hmgcr) involved in cholesterol biosynthesis. Srebp2, a cholesterol regulatory transcription factor was downregulated in het liver (Supplementary Fig. 4). Two major insulin-regulated transcription factors were found upregulated upon eIF6 depletion: Foxo1, suggesting a strong link between insulin signalling and eIF6-driven metabolic control and Pgc1-α (ref. 39), which is a key transcription factor involved in mitochondrial biogenesis and downregulated during lipogenesis (Supplementary Fig. 4c. Messenger RNAs differentially affected at the steady-state level by chronic eIF6 depletion, *in vivo*, were validated on multiple samples and ages (Fig. 4d; Supplementary Fig. 4b,c). For Fasn, Hmgcr, Pgc1-α and Foxo1 these changes were also confirmed at the protein level (Fig. 4e).

Next, we asked whether eIF6-induced changes were limited to the liver or also present in other insulin-responsive organs. White adipose tissue is controlled by systemic insulin. In contrast, the brain is unresponsive to systemic insulin due to the blood–brain barrier. eIF6 depletion induced co-ordinated changes in gene expression in fat and liver, but not in the brain (Fig. 4b; Supplementary Data 3). Intriguingly, eIF6 het fat and liver shared the downregulation of pathways regulating lipid and cholesterol synthesis (Fig. 4b,c). Muscle is also a major insulin responder. Analysis of liver, fat, muscle and brain at different ages showed that chronic eIF6 depletion caused a co-ordinated downregulation of Fasn mRNA and an increase in Pgc1-α mRNA^39^ in all insulin-responsive tissues, but not in brain (Supplementary Fig. 4c). Consistent trends were in general observed for muscle, fat and liver, but not brain for all genes studied with the notable exceptions of Zbtb16 that is liver specific and Foxo1, which was not changed in muscle (Supplementary Fig. 4c).

In conclusion, we show that impairment of translation, due to eIF6 depletion, causes a stable and co-ordinated change of gene expression of metabolic key-players at the mRNA level in insulin-responsive tissues, which leads to reduced lipogenesis. Thus, eIF6 is a translational regulator of metabolism, acting upstream of transcription.

### Fasn is a marker of eIF6 activation

**Figure 5:**
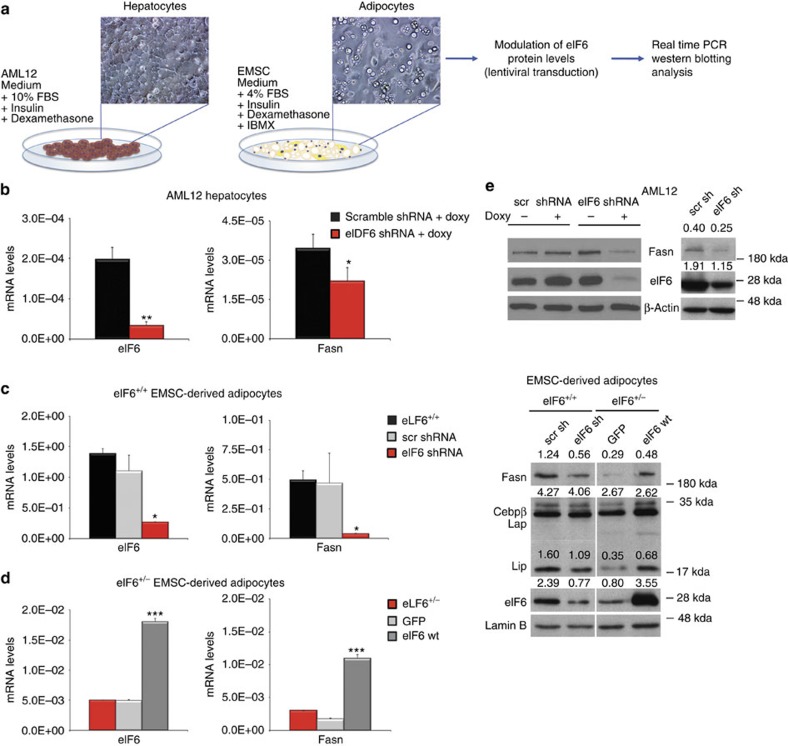
Fasn levels are controlled by eIF6 activity. (**a**) Rescue strategy in hepatocytes and primary stem cells. Cells were transduced with either eIF6 shRNA or wt eIF6, eIF6 protein levels were checked and Fasn mRNA and protein were analysed. (**b**) Downregulation of eIF6 by an inducible shRNA in AML12 hepatocytes leads to reduction of Fasn mRNA. (**c**–**d**) eIF6 has a direct effect on Fasn mRNA levels in mesenchymal stem cells differentiated into adipocytes. wt or het stem cells were either transduced with constitutive eIF6 shRNA (**c**) or wt eIF6 (**d**) and assayed for Fasn expression. A direct correlation between eIF6 levels and Fasn levels is observed. (**e**) Fasn protein is controlled by eIF6 levels in AML12 and EMSC adipocytes. Experiments on EMSC show both downregulation of eIF6 in wt cells or upregulation in het cells. Note eIF6 dependent levels of both Fasn and LIP isoform. In all panels, data are represented as mean±s.d. Panel **e** shows the action of two independent eIF6 shRNA, constitutive (right) or inducible. Statistical *P* values were calculated by two-tailed *t*-test (*P value ≤0.05, **P≤0.01, ***P≤0.001). Real-time experiments and western blotting were performed on biological triplicates of at least two independent experiments. One triplicate is shown for real time.

Fasn is a rate-limiting enzyme of fatty acid synthesis^40^, primarily induced by insulin. We demonstrated that its levels are downregulated by chronic eIF6 depletion in liver (Fig. 4) and that eIF6 induces the translation of lipogenic transcription factors. We therefore asked whether eIF6 could rapidly induce Fasn mRNA expression. We addressed the question in two models, liver-derived AML12 hepatocytes and mesenchymal stem cells (EMSC) differentiated to adipocytes. We either reconstituted or depleted eIF6 in these models (Fig. 5a). We found that depletion of eIF6 by using an inducible shRNA for eIF6 in AML12 hepatocytes led to a reduction of Fasn mRNA levels (Fig. 5b). eIF6 depletion led to a reduction of Fasn mRNA also in wt EMSC differentiated to adipocytes (Fig. 5c). Het EMSC differentiated to adipocytes express less eIF6 than wt cells. Next, we re-expressed eIF6 in het EMSC differentiated to adipocytes. Re-expression of eIF6 in het EMSC adipocytes restored Fasn mRNA levels (Fig. 5d).

Three alternative mechanisms may account for Fasn mRNA increase/decrease on eIF6 overexpression or depletion. First, variations in Fasn mRNA stability; second, effects of eIF6 expression on signalling pathways regulating transcription of Fasn mRNA, such as the mTOR pathway; third, translational regulation of transcription factors that shape fatty acid synthesis. To assess Fasn mRNA stability, we performed a time-course experiment after Actinomycin-D treatment in hepatocytes depleted for eIF6 and matched controls. We established that eIF6 depletion does not decrease Fasn mRNA stability (Supplementary Fig. 5a). mTOR controls both translation and lipogenic pathways. To rule out the possibility that eIF6 depletion impaired either the mTOR or the Akt pathway, we checked the phosphorylation state of both mTORc1 and mTORc2 substrates (although eIF6 is not downstream of mTOR activation). We found that eIF6 depletion, although generating a blunted insulin-translational response, does not induce changes in the phosphorylation of lipogenic pathways such as mTORc1, mTORc2 and ERK (Supplementary Fig. 5b). Also phosphorylation of IRS1, Akt, ERK, S6 and 4E-BP1 was not altered in the liver of het mice where eIF6 depletion is prolonged, consistent with insulin signalling unchanged by eIF6 (Supplementary Fig. 5b).

Finally, consistent with the hypothesis that eIF6 regulates Fasn transcription through the translation of transcription factors, depletion or overexpression of eIF6 simultaneously modulated both Fasn protein and C/EBPβ LIP isoform (Fig. 5e). Thus, Fasn expression can be used, in our system, as a model mRNA to test the efficacy of eIF6 inhibition.

### eIF6 targeting results in Fasn modulation

**Figure 6:**
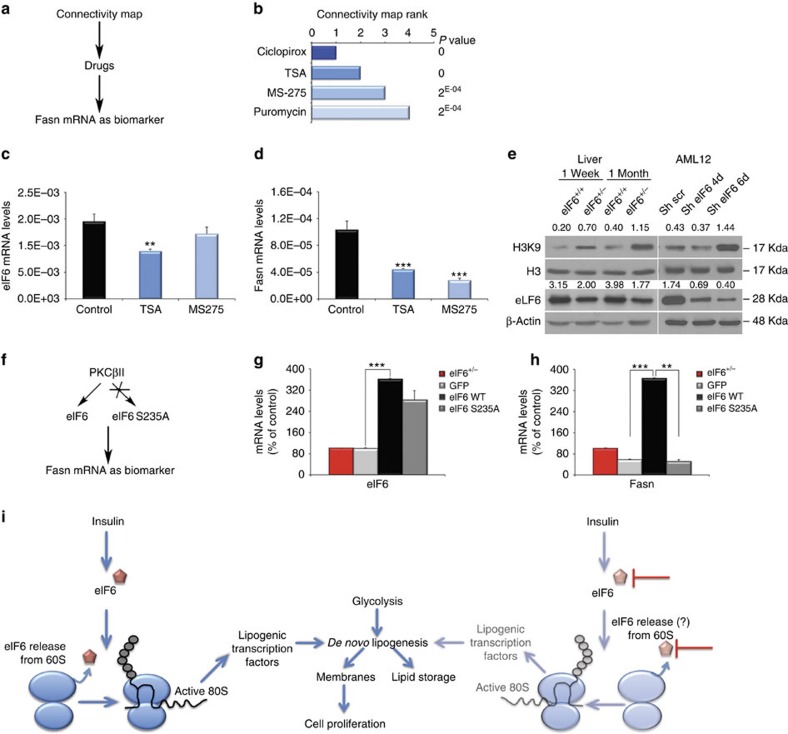
eIF6 targeting leads to Fasn reduction and histone acetylation. (**a**) Strategy through the connectivity map: drugs that generate a signature similar to eIF6 are identified and tested. (**b**) The connectivity map identifies two translational inhibitors, puromycin and ciclopirox and two HDAC inhibitors, TSA and MS-275. (**c**–**d**) HDAC inhibitors downregulate Fasn; HDAC inhibitor TSA, but not MS-275, reduces eIF6 mRNA (**c**), but both HDAC inhibitors downregulate Fasn mRNA (**d**). (**e**) Consistent with the Gene Ontology analysis (Fig. 4a) and the connectivity map, eIF6 depletion increases H3K9 acetylation. Western blot analysis of livers at indicated ages or AML12 cells. Representative blots with densitometry are shown. (**f**) Strategy through inhibition of eIF6 activation: mutation of eIF6 S235A is tested. S235A mutant is unable to restore translation following insulin stimulation^22^. (**g**–**h**) eIF6 reintroduction in EMSC cells induces Fasn. (**g**) Real-time analysis of restored wt and mutant eIF6 levels. (**h**) Analysis in the same cells of Fasn mRNA. wt eIF6 but not eIF6 S235A mutant, recovers Fasn mRNA levels. In all panels, data are represented as mean±s.d. Statistical *P* values were calculated by two-tailed *t*-test (*n*=3). Repeated at least twice. **P value ≤0.01, ***P≤0.001 (**i**) Model based on data of Figs 1, 2, 3, 4, 5, 6. Insulin induces translation. eIF6 favours translation of uORF-containing and G/C rich mRNAs encoding for lipogenic transcription factors. The net result is activation of the lipid biosynthetic pathway. eIF6 inhibition blocks lipogenesis and reverts insulin resistance.

Insulin-resistant conditions, such as obesity and type 2 diabetes, and cancer may arise from a common Fasn-dependent lipogenic state^40^. To discover possible functional connections between drugs action and eIF6 genetic perturbations, we analysed our microarray data through the Connectivity Map tool^41^, an online resource that enables the discovery of functional connections between drugs and genes through the feature of common gene-expression changes (Fig. 6a). We found that eIF6 depletion ‘signature' was similar to the one obtained by two translation inhibitors (Fig. 6b), ciclopirox (inhibiting eIF5A) and puromycin, binding the peptidyl transferase center (PTC) pocket in the large 60S subunit. eIF5A has been recently proposed to stimulate peptide-bond formation between proline residues^42^. Puromycin is an elongation inhibitor and one of the most potent inducer of termination of translation and peptide release. Other translational inhibitors like cycloheximide did not score significantly in this experiment. Two other top scorers were surprising, the histone deacetylase (HDAC) inhibitors, trichostatin A and MS-275 (Fig. 6b). HDAC inhibition has been recently suggested as effective in the treatment of type 2 diabetes^43^. We tested the hypothesis that HDAC inhibitors were able to modulate Fasn expression or eIF6 activity (Fig. 6c), as expected by the connectivity map. Indeed, 48 h treatment with TSA or MS-275 led to a reduction of Fasn mRNA (Fig. 6d). Short-term pulse with TSA or MS-275 did not alter initiation of translation (Supplementary Fig. 6). Since we found that chromatin remodelling appeared also in the Gene Ontology analysis, (see Fig. 4a), we tested the hypothesis that eIF6 regulates histone acetylation. Consistently with the connectivity map, eIF6 downregulation leads to histone hyperacetylation both in liver and in AML12 cells (Fig. 6e).

In several models, eIF6 activation requires phosphorylation of eIF6 Ser235 (refs 17, 22). Targeting eIF6 may be achieved using drugs that reduce eIF6 activation (Fig. 6f) by the PKC pathway^17^,^22^. Therefore, we expressed wt and mutant eIF6 in EMSC cells differentiated to adipocytes and measured eIF6 levels and Fasn expression. Figure 6g,h show that Fasn levels are not increased by Ser235Ala mutant eIF6, but only by wt eIF6.

In conclusion, either blocking eIF6 expression by HDAC inhibition or eIF6 activity by inhibition of its phosphorylation results in Fasn modulation providing proof-of-concept that eIF6 can be targetable. We conclude that the translational branch driven by insulin-eIF6 regulates long-term adaptation to high nutrient levels by eliciting glycolysis and lipogenesis (Fig. 6i).

## Discussion

We demonstrate that eIF6 translational activity regulates the lipogenic programme through the co-ordinated upregulation of enzymes involved in cholesterol and fatty acid synthesis. eIF6 acts in a cell autonomous fashion inducing the translation of lipogenic transcription factors like C/EBPβ, C/EBPδ and ATF4 characterized by rich G/C sequences or uORF in their 5′ UTR. The rate-limiting effect of eIF6 is evident after insulin stimulation. Thus, eIF6 activity links translation with metabolism affecting insulin sensitivity. The stimulatory effect of insulin on translation has been known for decades^44^; nonetheless, neither its physiological significance, nor its relevance for glycemic control is understood. By exploiting the finding that insulin-stimulated translation is blunted in eIF6-depleted cells, we demonstrate that insulin-stimulated translation is functional to the induction of transcriptional changes essential for the metabolic switch toward glycolysis and lipogenesis.

The switch to glycolysis and fatty acid synthesis induced by eIF6 in a cell autonomous fashion has implications in cancer. Indeed, the Warburg effect is a metabolic switch observed in cancer cells, where increased aerobic glycolysis is preferentially used as source of energy over oxidative phosphorylation. In addition, Fasn is a rate-limiting enzyme of *de novo* lipogenesis, a pathway needed to build membranes during oncogenesis^40^. Recently, we reported that eIF6 het mice were highly resistant to Myc-induced lymphomagenesis with up to 40% survival at 1 year. Myc is itself an inducer of glycolysis and protein synthesis^45^,^46^. Therefore, the anti-cancer role of eIF6 inhibition may be linked to its metabolic effect, counteracting Myc action. Transformed cells carrying the Ser235Ala eIF6 mutant, not toxic *in vitro*, cannot grow as xenografts. It follows that the mere hyperactivation of eIF6 and/or the translational machinery can explain part of the Warburg effect. Indeed, eIF6 is highly expressed in human aggressive tumours^47^, also by gene amplification^21^. Thus, our data further stress the powerful role that translation has in tumourigenesis^48^.

eIF6 facilitates the translation of mRNAs with G/C rich or uORFs sequences. We stress two facts. First, uORF-containing or G/C rich mRNAs are generally translated with poor efficiency. Second, eIF6 inhibition decreases also the rate of protein synthesis. It is the combination of qualitative and quantitative effects that results in a highly specific and powerful regulation of lipid biosynthesis, neither of them would be probably sufficient. It must be stressed that this conclusion is relevant, because insulin and growth factors always increase the rate of protein synthesis. The observation that uORFs inhibition is linked to reduction of lipogenesis in eIF6-depleted cells is somewhat unexpected, given the predominant model of their function in the UPR or amino-acid deficiency, but consistent with previous work showing that inhibition of eIF4G reduces loading of these mRNAs on polysomes^29^. We simply suggest that uORF can act as barriers for translation that can be overcome by translational stimulation. As such, they have been evolutionarily selected in mRNAs encoding for transcription factors that exploit high nutrient levels to build an anabolic response. Indeed, ATF4 role in triglycerides synthesis is clear^49^. To address the precise role of translation in the regulation of glucose and lipid metabolism, experiments of knockdown and rescue of uORFs sequences of these specific transcription factors *in vivo* are required. The genetic validation of translational control of metabolism will provide a new model for gene expression and metabolic control.

An unexpected observation is the maintenance of glucose tolerance, glucose uptake and glycogen synthesis in the presence of resistance to insulin-induced translation, observed in eIF6-depleted cells. In contrast, we see decreased glycolysis, fatty acid synthesis and ATP content. This fact underlines the specificity of the transcriptional programme elicited by eIF6 through translation. We provide a simple evolutionary model to explain our data. Glucose-stimulated insulin secretion signals to cells ‘richness' of nutrients. Cells convert this information in stimulation of glycolysis and fatty acid synthesis through the translational apparatus. Future studies are needed to investigate if the changes that we see in glucose and lipid metabolism could be related also to changes in energy charge. Our data fit a model in which eIF6-driven translation is a targetable feed-forward loop that induces insulin resistance and tumour growth. Therefore, we propose that translation, downstream of insulin, acts as a switch for ‘metabolic learning', that is, cells learn that they have nutrients (model, Fig. 6i)^50^.

The effect of eIF6 on translation is not a simple by-product of reduced ribosomal availability. First, knockout of 60S biogenesis factors, like Bop1, impairs ribosome production, but not Fasn levels (D.B., unpublished observations); second, eIF6 knockdown ‘signature' is similar only to puromycin but not to cicloheximide, another elongation inhibitor. Remarkably, puromycin and eIF6 are used together, *in vitro*, to dissociate post-termination ribosomes^51^. Our data would suggest that the anti-association activity of eIF6 is required to avoid inactive 80S formation after termination and allowing the rapid recycling of the free 40S on the adjacent start codon, *in vivo*.

The capability of translation, and eIF6, to steer metabolism has wide implications in diabetes. One surprising observation is that eIF6 inhibition is not pro-diabetic. In contrast, prolonged mTORc1-mediated inhibition of translation by rapamycin promotes diabetes onset^9^,^10^. It is unclear whether this effect is due to the pleiotropic effects of mTOR inhibition or whether this is due to the different step of translation inhibited by rapamycin. Rapamycin inhibits 4E-BPs phosphorylation, impairing the 48S formation step of initation, as well as elongation through eEF2 (ref. 7). eEF2 is linked to adaptation to nutrient deprivation^52^. Our model supports the specificity of eIF6-driven translation.

Our data bring further emphasis on some redundancy of insulin signalling pathways in cells. Good evidence shows that the fatty acid synthesis programme is activated by SREBP1 and highly stimulated by mTORc1 activity. Inhibition of lipogenesis is also a hallmark of mTORc1 inhibition^30^. We did not observe changes of SREBP1 at the transcriptional level or on polysomes (Supplementary Data 1-2). We found that the mTOR pathway is not altered by eIF6, as established by analysing pSer473-Akt, p235/236-S6 and 4E-BP1 phosphorylation status. Currently, there is no evidence that eIF6 is below the mTOR cascade. The hyperphosphorylated C terminus of eIF6 is not fully characterized^21^; the only characterized residue, phosphorylated by PKC, is Ser235 (ref. 20). PKC/RACK1 complex phosphorylates eIF6 contributing to its activation and tumour growth^53^ and RACK1 knockout mice have impaired PKC-stimulated translation^54^. In addition, PKCβII knockout have a lean phenotype and display resistance to obesity upon HFD^55^. However, in spite of not being under mTOR control, eIF6 regulates similar downstream processes, as demonstrated by our work. Therefore, the presence of an mTOR insensitive pathway, that through eIF6 controls lipogenesis, is consistent with the presence of alternative pathways converging on the regulation of metabolism. This is supported also by pharmacological evidence that mTOR inhibition can be bypassed by RAS mutations in cancer cells^13^.

In conclusion, we have demonstrated that translational control driven by eIF6 is sufficient to steer metabolic choices in an mTOR-independent fashion. We provide a mechanistic link between obesity and cancer through eIF6 activity.

## Methods

### Mouse colony

eIF6^+/+^ and eIF6^+/−^ mice were generated^22^ and backcrossed to C57BL/6N background (F⩾24). Metabolism experiments were performed also on a mixed 129 background with identical results. Both males and females were used. Experiments were performed with mice aged 1–3 months. The health status of mice was monitored every month according to the Federation of European Laboratory Animal Science Associations (FELASA) recommendations. Animals were genotyped and randomly analysed. There are no inclusion/exclusion criteria. Animal studies were performed on mice that were age-matched. Primary cells were always derived from littermates of the specified genotypes. Most measurements were performed automatically without knowing the genotype of the animals until analysis of data.

All experiments involving mice were performed in accordance to Italian national regulations and experimental protocols reviewed by the local Institutional Animal Care and Use Committees of the San Raffaele Research Institute (IACUC no. 477). After 2010, we complied to Directive 2010/63/EU based on three R principle.

Sample size was calculated by power analysis, set at minimum 85%. The variance is similar between the statistically compared groups. In all cases, and for all measurements at least three independent experiments each with at least two couples of matched experimental subjects were used. The primary phenotypic screening was performed on *n*=40 mice/genotype. Mice were fully randomized. Mice were housed with a 12 h light cycle (0700–1900 hours) and fed a standard rodent chow unless specified. A cohort of mice (*n*=20) was subject to HFD (27% safflower oil) regimen for 36 days. At the end, GTT and ITT were performed and insulin levels were measured.

### PCR analysis

Genotyping of the offspring mice was performed as follows. Briefly, PCR was performed by AmpliTaq Gold (Roche) according to the manufacturer's protocol. A shared primer number 3 (5′- GTGAGTCTGGCTTCATGTG -3′) was designed downstream of the deleted region. This primer can pair with wt allele-specific primer number 2 (5′- CTATGTGGCCTTGGTCCAC -3′) to amplify a 320 bp PCR product or with the targeted allele-specific primer number 1 (5′- GCAGCGCATCGCCTTCTATC -3′) in the neo cassette to amplify a 650 bp fragment from the mutant allele. PCR products were resolved on 1% agarose gel.

### GTT–ITT and clinical chemistry

For GTTs, randomized male mice (*n*=6) were fasted for 16 h and then injected intraperitoneally with 2 g kg^−1^ body weight glucose. Blood glucose values were determined by an automatic glucose monitor (Glucotrend 2, Roche) and Accu-Chek active bands (Roche)^56^. The ITT was performed on mice fasted for 4 h, human insulin was administered by IP injection at 1 U kg^−1^. Blood glucose levels were monitored as above immediately before injection (T0), and at 30, 60 and 120 min after injection. Blood was collected by retro-orbital puncture, centrifuged at 5,000*g* for 10 min and plasmas were used for the measurement of fasting blood glucose, cholesterol, triglycerides, non-essential fatty acids levels and markers of hepatic function by an Olympus AU400 chemistry analyzer.

### Cell cultures

We used 293T (ATCC CRL-3216) and AML12 (ATCC CRL-2254) cells. Cells were tested monthly for mycoplasma, and grown in Dulbecco's modified Eagle's medium/Ham's F-12 (GIBCO) supplemented with 10% foetal bovine serum, a mixture of insulin, transferrin and selenium (Invitrogen), 40 μg ml^−1^ dexamethasone and penicillin/streptomycin/glutamine solution (AML12) or fully supplemented as above but with Dulbecco's Modified Eagle's Medium (DMEM; 293T). After shRNA-carrying lentiviral vector infection(s), cells were selected in 5 μg ml^−1^ puromycin and shRNA was induced with 1 mg ml^−1^ doxycycline. For mRNA stability experiments, actinomycin-D 5 μg ml^−1^was added for 4, 8 and 24 h to AML12 cells. For drugs treatments, cells were treated with ciclopirox 15 μM, trichostatin A 100 nM, MS-275 10 μM and puromycin 7 μM for 30 min before polysome profile analysis or for 48 h for gene-expression analysis.

### Lentiviral infection

293T cells American Type Culture Collection (ATCC) were infected with packaging plasmid VSV-G, PMDLg/pRRE, pREV and transfer vector pCCL-PPT-hPGK^57^. Full-length wt or Ser235Ala mutant eIF6 and GFP were cloned in transfer vector. Primary hepatocytes were infected with eIF6wt-pCCL, eIF6S235A-pCCL and GFP-pCCL. Multiplicity of infection was determined to achieve twofold eIF6 expression by at least triplicate infections. For shRNA experiments, 293T cells were infected with packaging plasmid ENV, pMDG, pΔ8.74 and pGIPZ plasmids carrying scramble shRNA or two eIF6-specific shRNAs, defined after a screening of four sequences. For inducible shRNA of eIF6, pINDUCER11 plasmids kindly provided by Thomas Westbrook were used^58^. Mature antisense sequences were: 5′- AGCTTCCTACTAGCACCTG -3′; 5′- AGAAGTTCTCTGAGCCTCC -3′ (Open Biosystem).

### Dual luciferase assay

Plasmid transfections of AML12 cells at 50% confluency were performed by using calcium phosphate method. Cotransfections of 50% confluent AML12 were carried out in triplicate by using TK-ATF4-Luc fusion plasmids (kindly provided by Ronald C. Wek) and a Renilla luciferase plasmid serving as an internal control (Promega). After transfection (40 h), AML12 cells were treated with thapsigargin at 100 nM for 6 h or with no endoplasmic reticulum (ER) stress. Dual luciferase assays were carried out accordingly to Promega instruction manual after protein quantitation. Mean values o three independent experiments are expressed as the ratio of firefly versus renilla luciferase units (relative light units).

### Ear mesenchymal stem cell culture and adipogenesis

Mesenchymal stem cells from outer ears of eIF6^+/+^ and eIF6^+/−^ mice were collected and differentiated into the adipogenic lineage with DMEM-F12 supplemented with 4% foetal bovine serum, 1.7 μM insulin, 1 μM dexamethasone and 0.5 mM IBMX^59^ for 10 days. Differentiated cells were fixed in 10% buffered formalin and stained with Oil Red O for 10 min. The dye retained by the cells was eluted with isopropanol and quantified by measuring absorbance at 500 nm.

### Hepatocyte isolation

Three-months-old mice or young mice, up to 4 weeks, were anaesthetized by intraperitoneal injection of avertin. Liver was perfused *in situ* through the portal vein with two different perfusion media: T1 solution pH 7.4 (0.9% NaCl, 0.05% KCl, 0.2% HEPES and 0.08 mg ml^−1^ EGTA) and T2 solution pH 7.4 (0.6% NaCl, 0.05% KCl, 1.2% HEPES, 0.07% CaCl_2_ and 3 g ml^−1^ collagenase type I). Flow rate was 5 ml min^−1^. After collagenase digestion, hepatocytes were filtered through a 70 μm cell strainer and passed on a 37.5% PERCOLL cushion (Amersham). Pellet of viable cells was resuspended in DMEM-F12 and recovered at 5% CO_2_, 37 °C for 16 h before starting the experimental procedure^60^. Matched littermates were always analysed in parallel.

### Protein synthesis measurement by ^35^S-methionine labelling

Primary hepatocytes or AML12 hepatocytes were used for analysis of translational rate. Cells were seeded at sub-confluency in six-well plates, and insulin stimulation was performed at 100 nM for 30 min. Metabolic labelling was performed as described previously^22^.

### Glucose uptake

Glucose uptake was measured as the incorporation of radiolabeled 2-deoxyglucose^61^, in hepatocytes and 3T3-L1 differentiated adipocytes. Briefly, 2 × 10^5^ cells per well were grown in 12-well plates and serum-starved for 16 h. The cells were then washed twice with Krebs-Ringers Henseleit (KRH) buffer (20 mM HEPES pH 7.4, 136 mM NaCl, 4.7 mM KCl, 1.25 mM MgSO_4_ and 1.25 mM CaCl_2_) containing 0.1% bovine serum albumin (BSA) and incubated for 15 min in 0.45 ml KRH/BSA, followed by addition of 0.1 μM cold 2-deoxy-D-glucose and 0.5 μCi 2-deoxy-D-[^3^H]-glucose (Perkin Elmer) for 4 min. Following the incubation, cells were placed on ice, washed twice with ice-cold PBS and lysed in 0.5 ml of 0.05% sodium dodecyl sulfate (SDS). The lysate (0.4 ml) was counted in 5 ml of scintillation fluid using a Beckman LS6500 scintillation counter. Rates of non-specific glucose uptake were determined in samples pre-treated for 10 min with cytochalasin B (10 μM, Sigma) and were subtracted from the total uptake. The specific glucose uptake was normalized to protein content.

### Measurements of lactate secretion

Hepatocytes were plated at 2 × 10^5^ cells per well in 12-well dishes in high-glucose medium for 24 h (ref. 62). Cells were switched to serum-free high-glucose medium for 4 h. Lactate secreted into the medium was measured using the fluorescence mode of a Lactate Assay Kit (Biovision). Average of fluorescent intensity was calculated for each condition replicates. Values were normalized to protein content obtained from the same wells^30^.

### *De novo* lipogenesis

Hepatocytes were plated at 2 × 10^5^ cells per well in 12-well dishes in serum-free, high-glucose medium for 20 h. Cells were switched to serum-free, low-glucose (1 gr l^−1^) medium plus 4 μCi ml^−1^ D-[6-^14^C]-glucose (Perkin Elmer) for 4 h before lipid extraction. As a background control, one sample was labelled for just 1 min before extraction. Cells were washed twice with Dulbecco's phosphate-buffered saline (D-PBS) before lysing in 0.5% Triton X-100. The lipid fraction was extracted by sequential addition of chloroform and methanol (2:1 v/v) with vortexing. After washing with water, phase separation was achieved by low-speed centrifugation (1,000 r.p.m., 15 min). ^14^C incorporation into the lower lipid-containing phase was counted in 3 ml scintillation fluid. Each condition was assayed in triplicate. After subtracting background values, average readings were normalized to protein content^30^.

### Fatty acid oxidation

Liver were homogenized in sucrose-Tris-EDTA (STE) buffer containing 0.25 M sucrose, 10 mM Tris and 1 mM EDTA (or EGTA). The reaction mixture (100 mM sucrose, 10 mM Tris-HCl, 5 mM KH_2_PO_4_, 0.2 mM EDTA, 0.3% fatty acid-free BSA, 80 mM KCl, 1 mM MgCl_2_, 2 mM L-carnitine, 0.1 mM malate, 0.05 mM coenzyme A, DTT, ATP and [^14^C]-palmitate) was dispensed in each sample and incubated for 60 min at 37 °C. At the end of the incubation period, the reaction mixture was transferred to vials containing perchloric acid and highly basic filter paper in the cap and incubated for 1 h. Then, the filter paper disc, indicating the amount of CO_2_ liberated by fatty acid oxidation, was transferred to a glass scintillation vial with liquid scintillation counting cocktail. Average counts per minute (c.p.m.) were measured over 3 min (ref. 63).

### ATP content

Liver (200 mg) or AML12 cells were homogenized in 6% (v/v) ice-cold HClO_4_. Extracts were centrifuged at 10,000*g* for 10 min at 4 °C. The acid supernatant was neutralized with KCO_3_ and used for luminometric determination of ATP (ATP determination kit, Molecular Probes) by the method of Lundin^64^.

### Glycogen assay

Liver samples from 5 h-refed mice were homogenized in 6% perchloric acid (500 ml/100 mg tissue). Precipitates were pelleted and the supernatant was collected. One volume of H_2_O was added and the solution was adjusted to pH 6.5 with 10 N KOH. A fraction of each sample was then incubated with five volumes of amyloglucosidase (1 mg ml^−1^ in 0.2 M (pH 4.8) acetate buffer) at 40 °C for 2 h. Glucose concentrations were then determined using the Amplex Red Glucose/Glucose Oxidase Assay Kit (Invitrogen). Samples incubated in the absence of amyloglucosidase were used as baseline controls^62^.

### Histological staining

Tissue samples were excised from eIF6^+/+^ and eIF6^+/−^ mice and immediately fixed in 10% formaldehyde in 0.1 M phosphate buffer (PBS) at pH 7.4. Serial 3 μm sections of paraffin-embedded liver were stained with haematoxylin and eosin for morphological analysis or subjected to PAS staining. Briefly, sections were hydrated and oxidized in 0.5% periodic acid solution for 5 min. Then, slides are placed in Schiff reagent for 15 min. At the end of the procedure, sections were washed in tap water and counterstained with haematoxylin for 1 min. Stained samples were observed in a Zeiss Axiophot microscope and pictures were acquired with Scion Image Software.

### Polysomal profiles

Polysomal profiles were performed as follows. In brief, AML12 were lysed in 30 mM Tris-HCl, pH 7.5, 100 mM NaCl, 30 mM MgCl_2_, 0.1% NP-40, 10 mg ml^−1^ cycloheximide and 30 U ml^−1^ RNasin. The liver was gently homogenized in 50 mM Tris-HCl, pH 7.8, 240 mM KCl, 10 mM MgSO_4_, 5 mM DTT, 250 mM sucrose, 2% Triton X-100, 90 μg ml^−1^ cicloheximide and 30 U m^−1^ RNasin using a glass douncer. Heparin (100 μg ml^−1^) was added to the liver extract. After centrifugation at 12,000 r.p.m. for 10 min at 4 °C, cytoplasmic extracts with equal amounts of RNA was loaded on a 15–50% sucrose gradient and centrifuged at 4 °C in a SW41Ti Beckman rotor for 3 h 30 min at 39,000 r.p.m. Absorbance at 254 nm was recorded by BioLogic LP software (BioRad) and ten fractions (1.5 ml each) were collected for subsequent proteins and RNA extraction.

### mRNA extraction and real-time RT–PCR

Total RNA from tissue and cells was extracted with TRIzol reagent (Invitrogen). For total, subpolysomal and polysomal RNA extractions from sucrose gradient aliquotes, the sucrose fractions were divided in two. The pulled fractions 1–6 and 7–10 were used for subpolysomal and polysomal RNA. Afterward, samples were incubated with proteinase K and SDS 1% for 1 h at 37 °C. RNA was extracted by phenol/chloroform/isoamyilic acid method. After treatment of total RNA with RQ1 RNase-free DNase (Promega), reverse transcription was performed with Moloney Murine Leukemia Virus (MMLV) reverse-transcriptase enzyme (Promega) according to the manufacturer's instructions. Reverse transcribed complementary DNA (100 ng) was amplified with the appropriate primers. For liver microarray validation, Taqman probes specific for eIF6 (Mm04208296_m1), Fasn (Mm00662319_m1), Srebp1 (Mm00550338_m1), Srebp2 (Mm01306292_m1), Chrebp (Mm02342723_m1) Pgc1a (Mm01208835_m1), Foxo1 (Mm00490672_m1), Cdkn1a (Mm00432448_m1), Ccnb1 (Mm03053893_gH) and Zbtb16 (Mm01176868_m1) were used. Probes specific for syb green: GcK (Fw 5′- ctggatgacagagccaggatg -3′; Rw 5′- agttggttcctcccaggtct -3′); Pklr (Fw 5′- ctggatggggctgactgtat -3′; Rw; 5′- ggcgtagctcctcaaacaac -3′); Stearoyl-CoA desaturase 1 (Fw 5′- CTGACCTGAAAGCCGAGAAG -3′; Rw 5′- GCGTTGAGCACCAGAGTGTA -3′); Hmgcr (Fw 5′- TGTTCACCGGCAACAACAAGA -3′ Rw 5′- CCGCGTTATCGTCAGGATGA -3′); Lxra (Fw 5′- GAAGGAGAGAGCCTTGCGTA -3′ Rw 5′- ATTTGGTTGGGTCAACAAGG -3′); Lxrb (Fw 5′- GTTTCCAGGGCAACAGAGTC -3′ Rw 5′- CAGAGAACTTGTGGGGGAAG -3′) were used. Srebp1, Srebp2, Chrebp Target mRNA quantification by quantitative reverse-transcriptase PCR (RT–PCR) using ΔΔCt-method using Taqman Universal PCR Master Mix (4304437; Life Technologies, Invitrogen), with 18S rRNA as an internal standard, was performed on an ABIPRISM 7900HT Sequence Detection System (Applied Biosystems). For polysomal microarray validation, the following probes were used: Cebpd (Fw 5′- ATCGACTTCAGCGCCTACAT -3′, Rv 5′- GCTTTGTGGTTGCTGTTGAA -3′); Cebpb (Fw 5′- CAAGCTGAGCGACGAGTACA -3′ Rv 5′- AGCTGCTCCACCTTCTTCTG -3′). Target mRNA quantification by quantitative RT–PCR using ΔΔCt-method using SYBR Green Master Mix (4344463, Life Technologies, Invitrogen), with 18S rRNA as an internal standard, was performed on an ABIPRISM 7900HT Sequence Detection System (AppliedBiosystems). The data were expressed as the percentage of relative quantity of target genes. Results are represented as means+s.d. of three independent experiments.

### Microarray hybridization and bioinformatic analysis

Transcriptional profiling of murine tissue was performed with the use of Mouse Genome 430 2.0 Array (Affymetrix). Labelling reactions and hybridizations were carried out according to standard Affymetrix protocols (Affymetrix Genechip Fluidics Workstation). Raw microarray data were normalized using the Robust Multi-array Average algorithm^65^. Differentially expressed genes were detected using Significance Analysis of Microarrays^66^, a permutation based approach that allows the selection of a desired false discovery rate (FDR). Differentially expressed genes with an FDR <10% were deemed significant. Lists of differentially expressed genes have been functionally annotated using the web-based application DAVID^67^. A *P* value or EASE score (significance of gene–term enrichment with a modified Fisher's exact test) were calculated for each Gene Ontology term. A Benjamini–Hochberg FDR correction for multiple testing was applied and a threshold of FDR <10% was chosen for selecting significant enrichment of specific Gene Ontology terms or Kyoto Encyclopedia of Genes and Genomes pathways.

For microarray analysis on AML12, polysomal and subpolysomal RNAs were hybridized on the Agilent SurePrint G3 Mouse GE 8x60K Microarray G4852A following the manufacturer's protocol. The experiment was performed in biological quadruplicate. Hybridized microarray slides were scanned with an Agilent DNA Microarray Scanner G2505C at 3 μm resolution with the manufacturer's software (Agilent Scan Control 8.1.3). The scanned TIFF images were analysed numerically and the background was corrected using the Agilent Feature Extraction Software version 10.7.7.1 according to the Agilent standard protocol GE1_107_Sep09. The output of Feature Extraction was analysed with the R software environment for statistical computing (http://www.r-project.org/) and the Bioconductor library of biostatistical packages (http://www.bioconductor.org/). Low signal Agilent features, distinguished by a repeated ‘undetected' flag across the majority of the arrays in every condition, were filtered out from the analysis, leaving 18,905 features corresponding to 14,108 *MGI* genes. Signal intensities across arrays were normalized with the quantile method. Polysomal, subpolysomal and translational efficiency of differentially expressed genes (DEGs) were identified using linear models and moderated *t*-test as implemented in the Bioconductor Limma and tRanslatome packages (using a threshold of 0.05 on the Benjamini–Hochberg corrected *P* values). Translational efficiency was calculated as the ratio between polysomal and subpolysomal signals.

In addition to the standard procedure, for data on AML12, we include a differential analysis. Briefly, we modelled a correction filter that took in account the fact that polysomes of eIF6 shRNA and methionine incorporation were lower than in control cells (methionine incorporation het/wt 0.75; polysome area het/wt 0.6). We applied the correction to a selected number of targets, to predict their actual abundance, and verified it by digital RT–PCR. Wet validation confirmed that (relatively) upregulated mRNAs had similar (absolute) levels. We then include a calculation sheet with this interpretation.

UTR analysis: 5′ UTR sequences were downloaded from the UCSC Genome Browser (http://genome.ucsc.edu/), assembly GRCm38/mm10. For each *MGI* gene, the longest transcript variant was selected as representative of the gene. Distribution analysis was performed on the GC content of 5′ UTR regions of the lists of DEGs. All the distributions were compared with the background distribution corresponding to the whole set of mouse genes, and significant shifts were identified with the Mann–Whitney test.

### Connectivity map analysis

To detect transcriptional signatures overlapping with that of eIF6 haploinsufficiency, differentially expressed transcript IDs in the liver were converted to the Affymetrix U133A platform, ranked according to statistical significance and uploaded to the Connectivity Map (Broad Institute) tool^41^. The upregulated and downregulated genes in liver were compared with over 7,000 transcriptional signatures produced by *in vitro* treatment of cell lines with small bio-active molecules.

### Computed tomography method to determine liver fat content

Liver fat content was non-invasively determined by X-ray attenuation in computed tomography (CT) images (La Theta; Aloka Ltd., Tokyo, Japan) of the liver of sedated mice (continuous 2–2.5% isoflurane inhalation). A sagittal pre-scan was used to identify fixed anatomical landmarks to define a region from the proximal end of the eleventh thoracic vertebra to the distal end of the third lumbar vertebra. A polygonal region of interest in the ventral part of the liver was defined in three consecutive slices and analysed using the La Theta 2.10 software. Averages of computed tomography values of these region of interests were used to determine liver attenuation to estimate liver fat content.

### HOMA-IR analysis

A previously published and independent data set was used to confirm a clinical link between the expression of eIF6 and insulin sensitivity (GSE27949). This data set defines the basal transcriptional state of subcutaneous adipose tissue from 33 healthy subjects with ranging glucose tolerances. Their mean (s.d.) age, body mass index (BMI) and VO2max were 52.5 (11.7) years, 31.4 (7.3) kg m^−2^ and 45.5 (13.7) ml O_2_ per kg per minute. HOMA-IR was calculated for each participant by multiplying fasting glucose with fasting insulin dividing by 22.5. HOMA-IR was log transformed to approximate normality for regression analyses. Two CEL files were removed because they appeared to have technical issues based on quality diagnostics using the affyPLM Bioconductor package: GSM691122 and GSM691134. The affy package was then used to carry out RMA based normalization^68^,^69^. All analyses were performed using Bioconductor in R^69^.

### Western blotting and antibodies

SDS-PAGE were performed on protein extracts obtained from liver or hepatocytes homogenized in radioimmunoprecipitation assay buffer (RIPA) (10 mM Tris-HCl, pH 7.4, 1% sodium deoxycholate, 1% Triton X-100, 0.1% SDS, 150 mM NaCl and 1 mM EDTA, pH 8.0). The following antibodies were used: rabbit polyclonal antibody against eIF6 (1:3,000) (ref. 70). From Cell Signaling Technology antibodies were directed against: Fasn (1:1,000; #3180); acetyl-Histone H3 (Lys9) (1:1,000; #8173); Histone 3, (1:1,000; #4620); Foxo1 (1:1,000; L29) (#9454); phospho(Ser636/639)-IRS1 (1:1,000 #2388); phospho(Ser473)-AKT (1:1,000; #4060); AKT (1:1,000; #9272); phospho(Ser235/236)-S6 (1:1,000; #4858); S6 (1:1,000; #2217); 4E-BP1 (1:1,000; #9644); phospho(Thr202/Tyr204)-ERK (1:1,000; #4370); ERK (1:1,000; #9102); and phospho-(Thr638/641)-PKC beta (1:1,000; #9375). From other suppliers: PKC beta (1:1,000; C-18) sc-210 Santa Cruz Biotechnology; Cebpδ (1:500; M-17) sc-636 Santa Cruz Biotechnology; Pgc1α (1:500; H300) sc-13067 Santa Cruz Biotechnology; lamin B (1:1,000; M-20) sc-6217 Santa Cruz Biotechnology; Hmgcr (1:500; ABS229) Merck Millipore; C/EBPβ (1:500; 1Η7) 626002 Biolegend; β-actin (1:10.000; AC-15) A5441 Sigma; Lamin B (1:1,000; M-20) sc-6217 Santa Cruz Biotechnology.

Quantitation was performed on samples by running parallel lanes with replicates. Each lane was first normalized to β-actin and then normalized to the control sample. Relative expression ratios were obtained by densitometry using the ImageJ software. Uncropped images of the western blots membranes were shown in Supplementary Fig. 7.

### Statistical analysis

For comparing pair-wise genotypes on a single parameter, unless otherwise specified, results are given as the mean±s.d. Statistical *P* values calculated by two-tailed *t*-test were indicated: one asterisk for *P* values <0.05, two asterisks for *P* values <0.01, three asterisks for *P* values <0.001.

## Additional information

**Accession codes:** Microarray data have been deposited in GEO profiles under accession codes GSE61053 (wt and eIF6^+/−^ livers) and GSE61121 (we and eIF6^+/−^ fat and brain). Global gene-expression analysis of eIF6 shRNA and scramble shRNA in AML12 cells has been deposited in GEO under accession code GSE61126.

**How to cite this article:** Brina, D. *et al*. eIF6 coordinates insulin sensitivity and lipid metabolism by coupling translation to transcription. *Nat. Commun.* 6:8261 doi: 10.1038/ncomms9261 (2015).

## Supplementary Material

Supplementary Figures Supplementary Figures 1-7

Supplementary Data 1 Global analysis of eIF6 shRNA-infected and scramble shRNA infected AML12 cells. Data of subpolysomal (S) and polysomal (P) RNAs extracted from cells induced with doxycycline and treated 1 hour with insulin (DI). Raw signals sheet shows original data. Ratio normalization sheet shows ratio between polysomes and subpolysomes calculated taking in account either reduced polysome area or not.

Supplementary Data 2 Microarray analysis of wt and eIF6 +/- livers. Comprehensive analysis of microarray expression data in liver of eIF6 +/+ and +/- three weeks-old littermates. Raw data and statistical analysis. Raw signals sheet shows original data of both polysomal-associated and steady-state mRNAs. Statistical analysis of all changes between samples is shown in "Statistical analysis and genes". "Polysomal distribution" and "Transcriptionally regulated" referred to expression of polysome-associated and total mRNA.

Supplementary Data 3 Microarray analysis of wt and eIF6 +/- fat and brain. Comprehensive analysis of microarray expression data in fat and brain of eIF6 +/- and +/+ five weeks-old littermates. Raw data and statistical analysis are shown for each organ separately.
