# Supplementary material for: eIF6 coordinates insulin sensitivity and lipid metabolism by coupling translation to transcription
Source: Nat Commun. 2015 Sep 18;6:8261. doi: 10.1038/ncomms9261 (PMC4595657; doi:10.1038/ncomms9261)
Supplement: Supplementary Figures — 1-7 [file ncomms9261-s1.pdf]

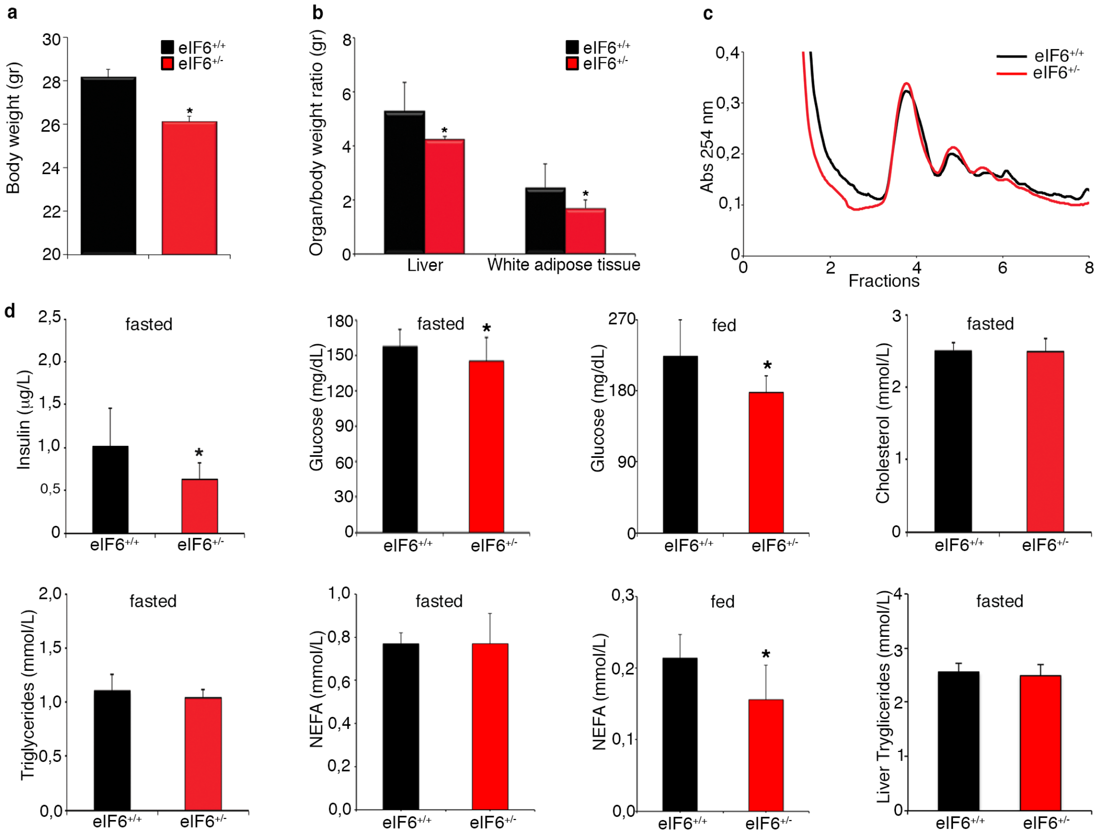

e

eIF6	female		male		ANOVA
	eIF6 +/+ n=10 mean ± sd	eIF6 +/- n=10 mean ± sd	eIF6 +/+ n=10 mean ± sd	eIF6 +/- n=9 mean ± sd	p-value
ALAT/GPT [U/l]	29 ± 20	33 ± 16	41 ± 31	31 ± 8	0.683
ASAT/GOT [U/l]	44 ± 11	49 ± 11	58 ± 11	62 ± 18	0.286
alpha-Amylase [U/l]	554.49 ± 71.71	554.2 ± 74.96	668.74 ± 59.93	669.1 ± 64.9	0.999
LDH [U/l]	230 ± 83.6	274.4 ± 130.7	256.6 ± 55.2	262.3 ± 81.7	0.403
Total proteine [g/l]	48.4 ± 2.4	49.3 ± 1.9	51.9 ± 0.7	51.7 ± 1.3	0.473
Albumin [g/l]	26.2 ± 1.8	27 ± 1.4	26.8 ± 0.4	27.3 ± 0.9	0.134
Creatinine [µmol/l]	9.94 ± 1.17	9.94 ± 1.31	8.7 ± 1.12	9.45 ± 1.37	0.353
Urea [mmol/l]	10.4 ± 1.32	10.84 ± 1.44	11.13 ± 1.23	10.71 ± 1.48	0.979
Sodium [mmol/l]	148 ± 2	148 ± 2	154 ± 1	153 ± 3	0.711
Potassium [mmol/l]	3.8 ± 0.4	3.8 ± 0.5	4.1 ± 0.4	4.3 ± 0.4	0.618
Chloride [mmol/l]	109.4 ± 2	109.9 ± 1.8	109.8 ± 0.9	109.5 ± 1.5	0.823
Calcium [mmol/l]	2.31 ± 0.06	2.36 ± 0.08	2.45 ± 0.08	2.41 ± 0.06	0.711
Inorganic phosphate [mmol/l]	1.6 ± 0.5	1.5 ± 0.4	1.4 ± 0.4	1.2 ± 0.3	0.218
Iron [µmol/l]	30.7 ± 3.8	27 ± 4.3	22.6 ± 2.1	24.2 ± 1.3	0.31
ALP [U/l]	135 ± 12	143 ± 11	102 ± 9	93 ± 5	0.934

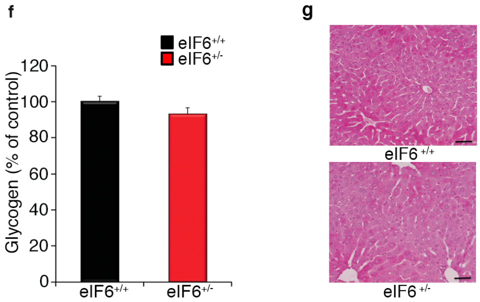

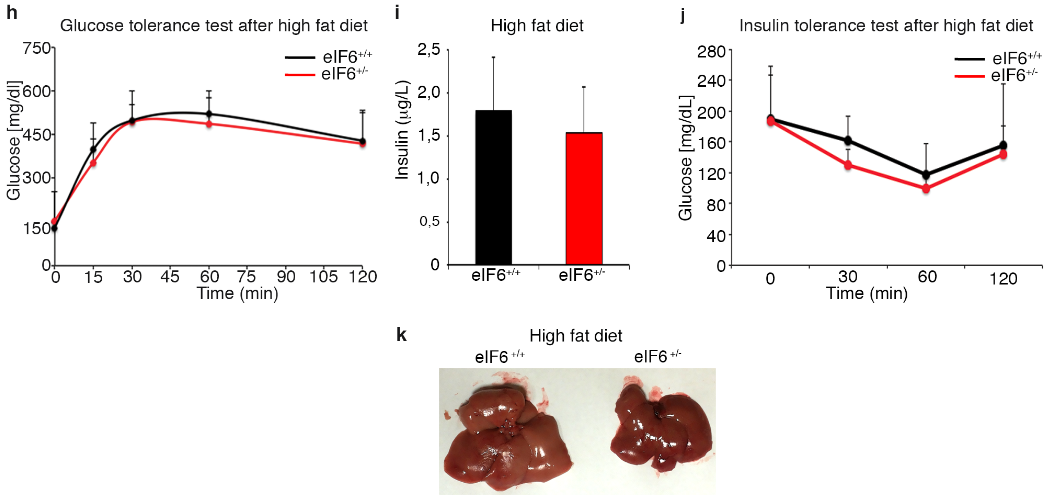

Supplementary Figure 1

Supplementary Fig. 1 eIF6 +/- mice show a reduction in white adipose tissue, blood lipids and normal glycogen synthesis. The cohort of the original phenotypic screening was n=40. For specific tests, the actual number of animals which have been analysed is included. Data are represented as mean ± SD. Statistical p-values were calculated by two-tailed t-test (*, p-value≤ 0.05) **a-b**) eIF6 het. mice are leaner; **a**) body weight (males) and (**b**) liver and fat mass of eIF6 +/- mice compared to +/+. (**c**) Fasting polysomes of eIF6 +/- mice compared to +/+ show no differences. Representative polysomal profiles of liver from overnight fasted mice display no differences in polysomes as well as high 80S in both genotypes. Experiment repeated at least five times. (**d**) Blood analysis of insulin, glucose, cholesterol, triglycerides and NEFA in the defined conditions. Experimental samples, n=10, sex: males. **(e)** Blood enzymes and electrolytes (liver markers) of the eIF6 +/- cohort used in the study. N indicated in table. **(f-g)** Liver glycogen depots are not affected by eIF6 haploinsufficiency as quantified by enzymatic analysis (f). PAS (glycogen) staining in the liver of eIF6 +/+ (up) and +/- het (bottom) mice after feeding shows no differences (g). Scale bar, 50 μM. The analysis was performed on 6 mice. (**h**) Glucose tolerance test performed after high fat diet is comparable between eIF6 +/+ and +/- het mice. N=9. **(i)** Insulin levels at time 0 of the GTT. **(j)** Insulin tolerance test shows a faster reduction of glucose in het mice. N=5 **(k)** Representative images of livers of eIF6 wt and het mice after high fat diet.

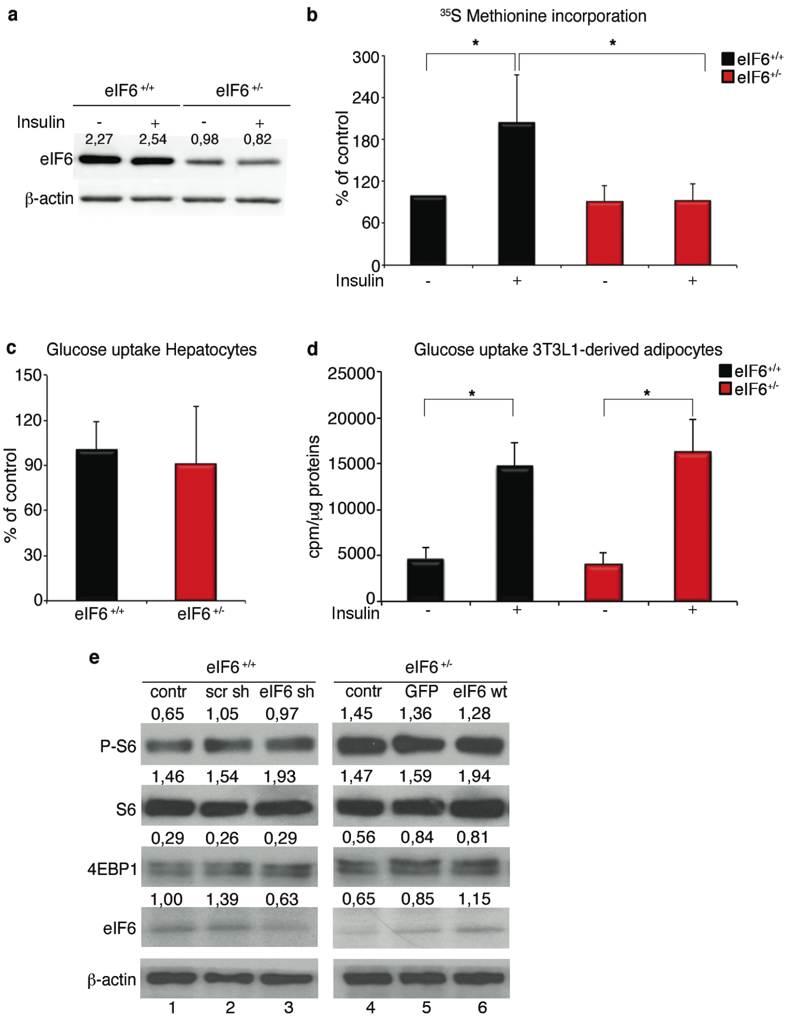

Supplementary Figure 2

Supplementary Fig. 2 eIF6 heterozygous cells show reduced translation in response to insulin, but normal glucose uptake and mTORc1 activation. (**a**) Western blot analysis showing eIF6 levels. The experiment has been performed more than 5 times. (**b**) ^{35}S-methionine labeling in isolated primary hepatocytes stimulated with 100 nM insulin. The basal rate of translation is indicated as 100%. Statistical p-values were calculated by two-tailed t-test by comparing primary cells from two littermates, quantitated in triplicate. Normalization was performed on total protein content. The experiment has been repeated on N=3 littermate couples. (**c**) Insulin-stimulated glucose uptake in primary hepatocytes. N=6 (biological). **(d)** Insulin-stimulated glucose uptake in 3T3-L1 adipocytes with or without eIF6 shRNA. N=6 (biological). (**e**) Phosphorylation of Ser235/236-S6 and 4E-BP1 is identical in eIF6 +/+ hepatocytes and eIF6 +/+ depleted for eIF6, as well as in eIF6 +/- hepatocytes reconstituted with eIF6. Depletion and reconstitution was performed on primary hepatocytes infected with lentiviral vectors. The blot on the left with eIF6 +/+ cells and the blot on the right with eIF6 +/- cells cannot be compared to each other because they have been performed at different times, on different primary hepatocytes and with different lentiviral backbones, as indicated. Note that lentiviral infection induces acutely, to some extent, eIF6. Representative data. The experiment (WB) has been repeated N≥4. The specific blot relates to Fig. 2d-e.

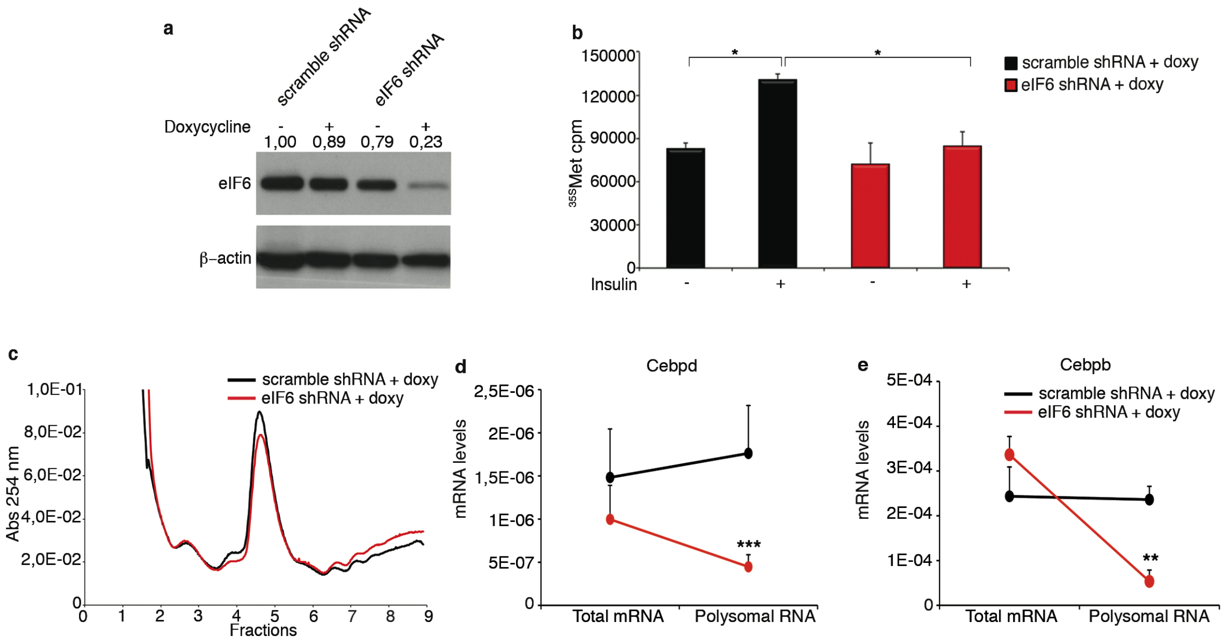

Supplementary Figure 3

Supplementary Fig. 3 AML12 infected with shRNA specific for eIF6 display reduction of methionine incorporation and reduction of polysome-associated lipogenic transcription factors like CEBPβ and CEBPδ, after insulin stimulation (**a**) eIF6 protein levels after 48 hours of doxycycline treatment compared to scramble shRNA-infected AML12. Representative blot. The experiment has been performed N≥8. (**b**) Metabolic labeling after 100 nM insulin stimulation for 1 hr. Values as c.p.m. Data are represented as mean ± SD. Statistical p-values were calculated by two-tailed t-test (n = 2. *, p-value≤ 0.05). Normalization was performed on total protein content. Representative experiment, technical triplicates. The experiment has been performed other two times with freshly infected cells **(c)** Polysome profile from unstimulated (without insulin) AML12 cells with eIF6 shRNA show that they are identical to scramble controls. Specifically, this is the unstimulated control of Fig. 3A. (**d**) Real time validation of expression of the 5'G/C-rich mRNAs of Cebpδ, a key transcription factor involved in lipid metabolism, downregulated on polysomes of eIF6 depleted cells, compared to control. (**e**) Real time validation of expression of the uORF-containing mRNAs of Cebpβ, a key transcription factor involved in lipid metabolism, downregulated on polysomes of eIF6 depleted cells, compared to control. (d) and (e) are from N=3.

a

gene_assignment	p-value	Total Fold-Change
NM_010579 // Eif6 // eukaryotic translation initiation factor 6	1,8789E-03	-1,79871

Glucose and lipid metabolism		Total
**gene_assignment**	**p-value**	**Fold-Change**
NM_009657 // Aldoc // aldolase C, fructose-bisphosphate	8,2514E-03	-2,29691
NM_013631 // Pklr // pyruvate kinase liver and red blood cell	2,2400E-03	-2,59668
NM_011044 // Pck1 // phosphoenolpyruvate carboxykinase 1	1,1514E-02	3,08253
NM_030210 // Aacs // acetoacetyl-CoA synthetase	2,3366E-03	-8,6172
NM_133360 // Acaca // acetyl-Coenzyme A carboxylase alpha	2,7605E-03	-1,62465
NM_133904 // Acacb // acetyl-Coenzyme A carboxylase beta	2,8101E-03	-3,92506
NM_134037 // Acly // ATP citrate lyase	6,0156E-03	-2,22097
NM_028817 // Acsl3 // acyl-CoA synthetase long-chain family member 3	2,3411E-03	-2,14071
NM_019811 // Acss2 // acyl-CoA synthetase short-chain family member 2	6,7593E-05	-3,63512
NM_198636 // Acss3 // acyl-CoA synthetase short-chain family member 3	4,4969E-03	-1,58614
NM_019422 // Elovl1 // elongation of very long chain fatty acids	5,4595E-04	-2,20779
NM_130450 // Elovl6 // ELOVL family member 6, elongation of long chain fatty acid	3,8054E-02	-3,11512
NM_007988 // Fasn // fatty acid synthase	5,1772E-03	-2,32226
NM_008255 // Hmgcr // Hmg-Coenzyme A reductase	1,3803E-02	-2,67554
NM_145942 // Hmgcs1 // Hmg-Coenzyme A synthase 1	1,6185E-02	-2,82391
NM_008290 // Hsd17b2 // hydroxysteroid (17-beta) dehydrogenase 2	3,1171E-03	-1,66173
NM_010476 // Hsd17b7 // hydroxysteroid (17-beta) dehydrogenase 7	1,3873E-02	-2,90274
NM_019657 // Hsd17b12 // hydroxysteroid (17-beta) dehydrogenase 12	1,7508E-04	-1,82023
NM_008615 // Me1 // malic enzyme 1, NADP(+)-dependent	5,2268E-04	-2,47926
NM_023556 // Mvk // mevalonate kinase	4,8311E-03	-1,95486
NM_026784 // Pmvk // phosphomevalonate kinase	3,3126E-03	-2,00947
NM_009127 // Scd1 // stearoyl-Coenzyme A desaturase 1	6,7779E-02	-4,90359
NM_028881 // Crtc2 // CREB regulated transcription coactivator 2	2,6730E-02	1,55426
NM_019739 // Foxo1 // forkhead box O1	1,1037E-03	2,06985
NM_008904// Ppargc1a // perox prolact rec g coactivator 1a	2,1833E-03	3,9795

Cell cycle		Total
**gene_assignment**	**p-value**	**Fold-Change**
NM_007631 // Ccnd1 // cyclin D1	4,2974E-02	-1,44027
NM_007633 // Ccne1 // cyclin E1	2,1941E-03	-1,62902
NM_009828 // Ccna2 // cyclin A2	3,9281E-04	-3,5108
NM_172301 // Ccnb1 // cyclin B1	1,0550E-04	-5,28301
NM_007630 // Ccnb2 // cyclin B2	1,9420E-03	-2,40688
NM_007659 // Cdc2a // cell division cycle 2 homolog A	2,7368E-04	-2,33774
NM_011497 // Aurka // aurora kinase A	5,0716E-06	-2,83064
NM_011496 // Aurkb // aurora kinase B	7,0752E-04	-2,22977
NM_011121 // Plk1 // polo-like kinase 1	5,1683E-06	-3,44378
NM_007900 // Ect2 // ect2 oncogene	3,3000E-05	-4,27353
NM_007669 // Cdkn1a // cyclin-dependent kinase inhibitor 1A	2,0174E-03	2,61568
NM_001033324 // Zbtb16 // zinc finger and BTB domain containing 16	1,8602E-06	7,8393

Histones		Total
**gene_assignment**	**p-value**	**Fold-Change**
NM_030609 // Hist1h1a // histone cluster 1, H1a	0,0054943	-2,26323
NM_020034 // Hist1h1b // histone cluster 1, H1b	0,0022563	-2,66809
NM_013550 // Hist1h3a // histone cluster 1, H3a	0,0004817	-2,05066
NM_178215 // Hist2h3b // histone cluster 2, H3b	0,0007131	-2,50599
NM_178216 // Hist2h3c1 // histone cluster 2, H3c1	0,0037202	-2,07224

b

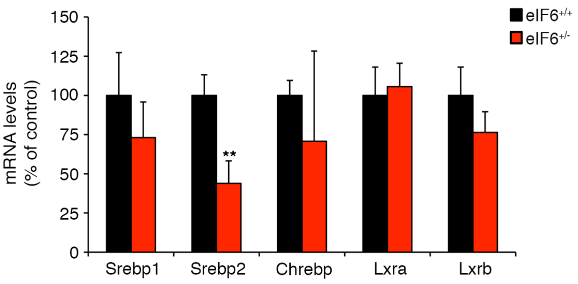

c

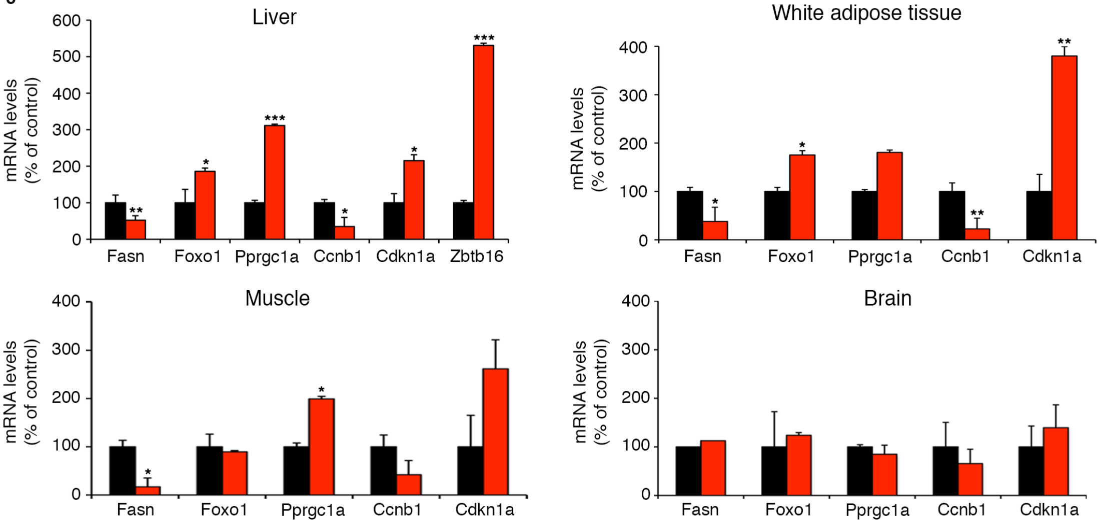

Supplementary Figure 4

Supplementary Fig. 4 Overview of three major pathways affected by eIF6. Fasn mRNA is downregulated in all insulin responsive tissues of eIF6 +/- mice. (**a**) Overview of representative genes differentially expressed in liver of eIF6 +/- and +/+ littermates underline coordinated changes in gene transcription (see also Supplementary Table 2 for full list). (**b**) Quantitative PCR for other transcription factors involved in metabolism in the liver. Of these, only Srebp2, important for cholesterol synthesis, is affected by eIF6 expression 4d. (**c**) Real-time analysis of several targets involved in metabolism and cell cycle progression in liver, fat, mucle and brain. Fasn (fatty acid synthase) and Pgc1α (PPARγ coactivator 1α) show specific changes in insulin-responsive tissues,liver, fat and muscle. Note that gene expression in the brain does not change, consistently with the Heathmap (Figure 4b). Fasn levels are downregulated in all three tissues suggesting a dependence from eIF6 levels. The overall gene signature indicates that eIF6 deficiency reduces lipid synthesis and cell cycle progression. Data are represented as mean ± SD. Statistical p-values were calculated by two-tailed t-test (*, p-value≤ 0.05). Data represented come from technical triplicates (three reactions) of biological triplicates analysed (three animals/genotype). The experiment has been performed at multiple ages of mice with similar results.

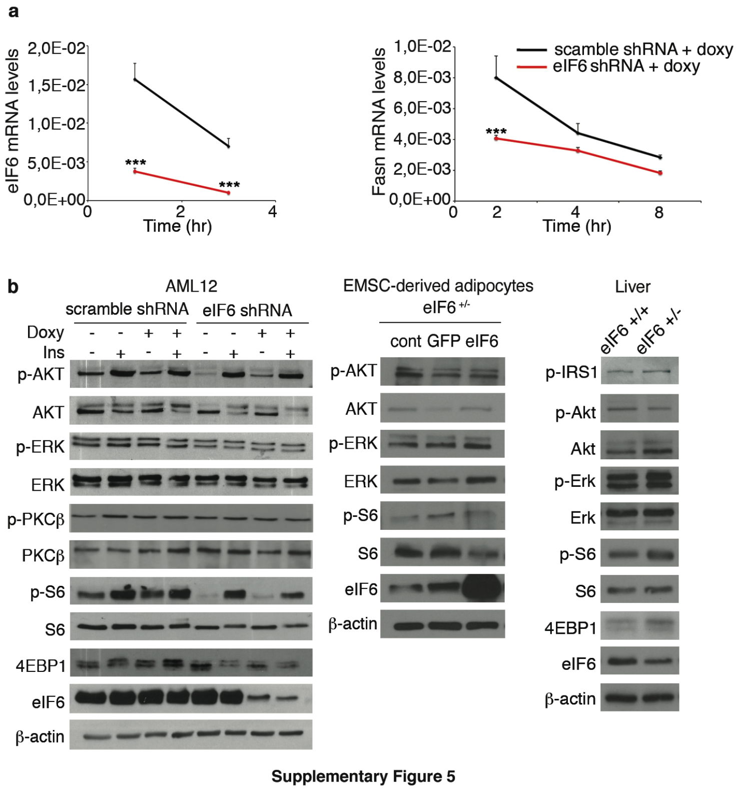

Supplementary Fig. 5 Fasn levels are not regulated at the level of mRNA stability or signaling pathways: mTOR/PKC activity in eIF6-depleted cells is normal. (**a**) Fasn mRNA stability was tested in AML12 hepatocytes in which eIF6-specific or scramble shRNA was induced for 6 days by 1 µg/ml doxycycline. 5 µg/ml actinomycin D were added for 0, 4 and 8 hr and eIF6 (left panel) and Fasn levels (right panel) were quantitated with Qt-PCR (left panel). eIF6 does not affect Fasn mRNA stability Data are represented as mean ± SD. Statistical p-values were calculated by two-tailed t-test (n = 2. ***, p-value ≤ 0.001). Technical triplicates of an experiment performed on two biological replicates, in different days are shown. (**b**) The activation of mTOR pathway in response to insulin is not affected by eIF6 depletion. Phosphorylation of AKT (Ser473), ERK (Thr202/Tyr204), S6 (Ser235/236) and 4EBP1 is not changed upon eIF6 silencing in AML12 hepatocytes (left panel). eIF6 re-expression in eIF6 +/- EMSC-derived adipocytes does not change the phosphorylation state of representative mTORc1/mTORc2/MAPK cascade targets (middle panel). Right panel shows the indicated proteins in a representative blot from refed livers. Note eIF6 protein levels consistent with shRNA and heterozygosity. Blots have been performed for each antigen at least four times (biological replicates).

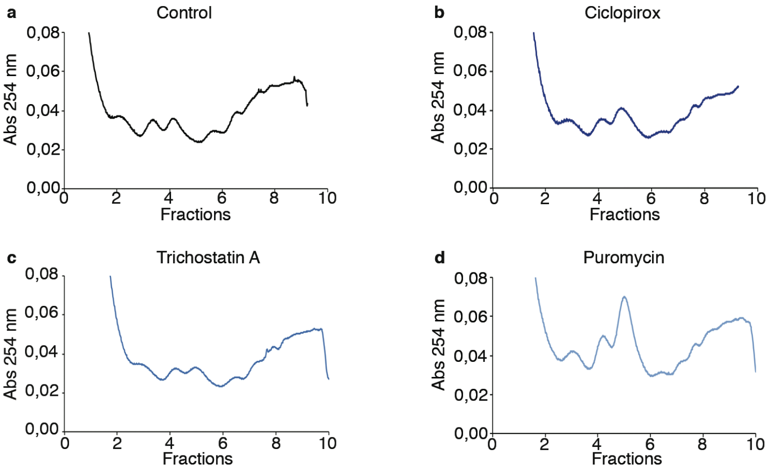

Supplementary Figure 6

Supplementary Fig. 6 Polysomal profiles of drugs identified by the Connectivity map. (a-d) Representative polysome profiles performed in untreated AML12 of after short-term treatment (30 minutes) with ciclopirox15 μm, trichostatin A 100 nM and puromycin 7 μM. Puromycin induces a severe increase in 80S peak and a slight decrease in polysomes, while ciclopirox has marginal effects on polysome profile. Trichostatin A does not affect translation. See text for explanations.

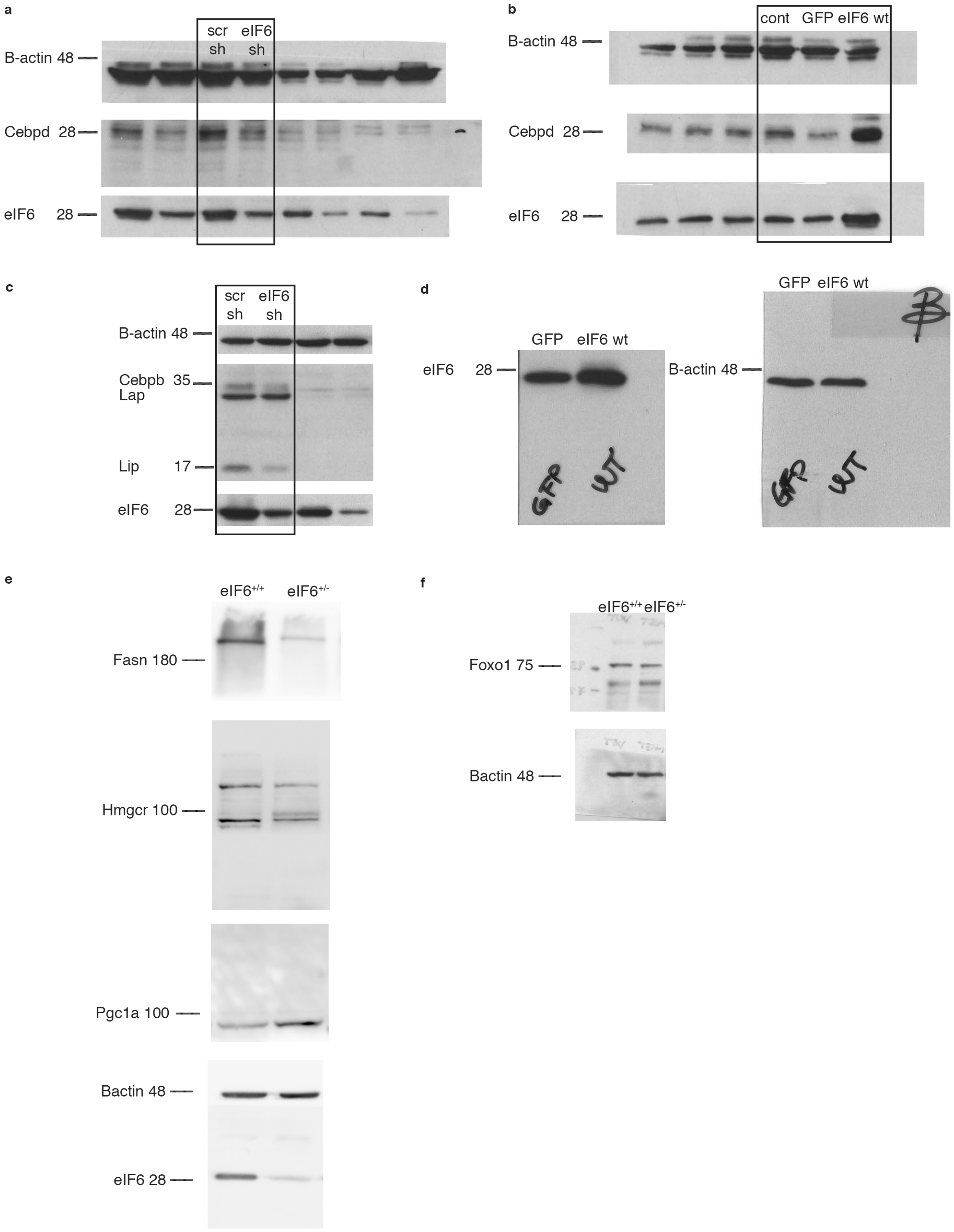

Supplementary Fig. 7 Uncropped western Blots. (a-b) Figure 3f. **(c)** Figure 3g. **(d)** Figure 3h. **(e-f).** Figure 4e.
